# Improvement of Fusel Alcohol Production by Engineering of the Yeast Branched-Chain Amino Acid Aminotransaminase

**DOI:** 10.1128/aem.00557-22

**Published:** 2022-06-14

**Authors:** Jirasin Koonthongkaew, Nontawat Ploysongsri, Yoichi Toyokawa, Vithaya Ruangpornvisuti, Hiroshi Takagi

**Affiliations:** a Division of Biological Science, Graduate School of Science and Technology, Nara Institute of Science and Technology, Ikoma, Nara, Japan; b Department of Chemistry, Faculty of Science, Chulalongkorn University, Patumwan, Bangkok, Thailand; Kyoto University

**Keywords:** *Saccharomyces cerevisiae*, yeast, branched-chain amino acid, branched-chain higher alcohol, branched-chain amino acid aminotransaminase, Bat1, Bat2, *in silico* computational analysis

## Abstract

Branched-chain higher alcohols (BCHAs), or fusel alcohols, including isobutanol, isoamyl alcohol, and active amyl alcohol, are useful compounds in several industries. The yeast Saccharomyces cerevisiae can synthesize these compounds via the metabolic pathways of branched-chain amino acids (BCAAs). Branched-chain amino acid aminotransaminases (BCATs) are the key enzymes for BCHA production via the Ehrlich pathway of BCAAs. BCATs catalyze a bidirectional transamination reaction between branched-chain α-keto acids (BCKAs) and BCAAs. In S. cerevisiae, there are two BCAT isoforms, Bat1 and Bat2, which are encoded by the genes *BAT1* and *BAT2*. Although many studies have shown the effects of deletion or overexpression of *BAT1* and *BAT2* on BCHA production, there have been no reports on the enhancement of BCHA production by functional variants of BCATs. Here, to improve BCHA productivity, we designed variants of Bat1 and Bat2 with altered enzyme activity by using *in silico* computational analysis: the Gly333Ser and Gly333Trp Bat1 and corresponding Gly316Ser and Gly316Trp Bat2 variants, respectively. When expressed in S. cerevisiae cells, most of these variants caused a growth defect in minimal medium. Interestingly, the Gly333Trp Bat1 and Gly316Ser Bat2 variants achieved 18.7-fold and 17.4-fold increases in isobutanol above that for the wild-type enzyme, respectively. The enzyme assay revealed that the catalytic activities of all four BCAT variants were lower than that of the wild-type enzyme. Our results indicate that the decreased BCAT activity enhanced BCHA production by reducing BCAA biosynthesis, which occurs via a pathway that directly competes with BCHA production.

**IMPORTANCE** Recently, several studies have attempted to increase the production of branched-chain higher alcohols (BCHAs) in the yeast Saccharomyces cerevisiae. The key enzymes for BCHA biosynthesis in S. cerevisiae are the branched-chain amino acid aminotransaminases (BCATs) Bat1 and Bat2. Deletion or overexpression of the genes encoding BCATs has an impact on the production of BCHAs; however, amino acid substitution variants of Bat1 and Bat2 that could affect enzymatic properties—and ultimately BCHA productivity—have not been fully studied. By using *in silico* analysis, we designed variants of Bat1 and Bat2 and expressed them in yeast cells. We found that the engineered BCATs decreased catalytic activities and increased BCHA production. Our approach provides new insight into the functions of BCATs and will be useful in the future construction of enzymes optimized for high-level production of BCHAs.

## INTRODUCTION

The branched-chain higher alcohols (BCHAs), i.e., isobutanol (IUPAC: 2-methylpropan-1-ol), isoamyl alcohol (IUPAC: 3-methylbutan-1-ol), and active amyl alcohol (IUPAC: 2-methyl-1-butanol), belong to a group of alcohols with high aliphaticity that are formed from the degradation products of branched-chain amino acids (BCAAs; e.g., valine [Val] degradation products to form isobutanol, leucine [Leu] into isoamyl alcohol, and isoleucine [Ile] into active amyl alcohol). BCHAs have elicited broad interest as potential next-generation biofuels to replace ethanol, because they have a higher energy density and octane number and a lower hygroscopicity than ethanol (1, 2). In addition, BCHAs are widely used in several industries as solvents and extractants for organic compounds (3). Within the food industry, BCHAs and their acetate derivatives are well known as important flavor compounds in several beverages (4–6), fermented foods (soy sauce, fermented milk, and cheeses) (7, 8), and bread (9). With such a wide array of benefits in several industries, the demand for BCHAs is continuously rising (China’s market is 500,000 tons/year for isobutanol and more than 10,000 tons/year for each of isoamyl alcohol and active amyl alcohol). BCHAs have been traditionally produced from petrochemical pathways (10). However, due to environmental and sustainability concerns, microbial production of BCHAs is an attractive alternative approach (1, 11).

**FIG 1:**
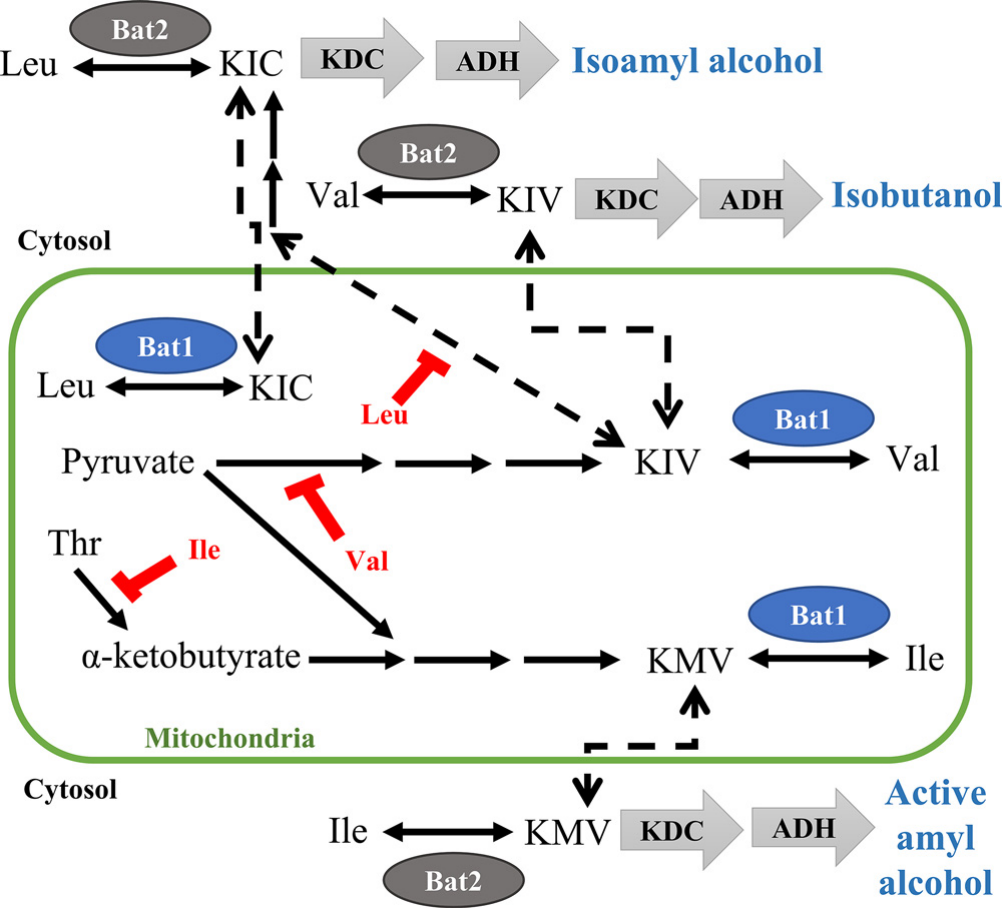
Schematic metabolism of branched-chain amino acids (BCAAs) and branched-chain α-keto acids (BCKAs) in S. cerevisiae. α-Ketoisovalerate (KIV) and α-keto-β-methylvalerate (KMV) are mainly synthesized in mitochondria from two pyruvates for KIV or one pyruvate and α-ketobutyrate for KMV. α-Ketoisocaproate (KIC) is produced from KIV by the reaction occurring in mitochondria and cytoplasm. KIV, KIM, and KIC are then transaminated to valine (Val), isoleucine (Ile), and leucine (Leu), respectively, by branched-chain amino acid aminotransaminases (BCATs) localized in the mitochondria (Bat1) or cytoplasm (Bat2). Bat1 and Bat2 can also catabolize BCAAs to BCKAs by the reaction of oxidative deamination in the cytoplasm or mitochondria. BCKAs (KIV, KMV, and KIC) are further converted into branched-chain higher alcohol (BCHAs; isobutanol, active amyl alcohol, and isoamyl alcohol, respectively) by the reaction of α-keto acid decarboxylases (KDCs) and alcohol dehydrogenases (ADHs). The biosynthetic enzymes, Ilv6, Ilv1, and Leu4/Leu9, are subject to feedback inhibition by Val, Ile, and Leu, respectively (shown in red).

The yeast Saccharomyces cerevisiae has attracted interest as a better host for BCHA production than bacteria, because it has a higher isobutanol tolerance than bacteria (up to 20 g/L) and naturally produces a small amount of these compounds during fermentation (12–14). In S. cerevisiae, BCHAs are synthesized from the metabolic pathways of BCAAs (Val, Leu, and Ile) through the key intermediates, branched-chain α-keto acids (BCKAs) (Fig. 1). The biosynthesis of BCAAs mainly takes place in mitochondria. Val and Ile are synthesized from parallel reactions, starting from two molecules of pyruvate (for Val) or one pyruvate and one α-ketobutyrate (for Ile). The sequential reactions occur and convert two pyruvates or one pyruvate and one α-ketobutyrate into the key intermediate BCKAs: α-ketoisovalerate (KIV) and α-keto-β-methylvalerate (KMV) for Val and Ile, respectively (15). For Leu biosynthesis, KIV undergoes the alternative reaction sequences and transport out of mitochondria into α-ketoisocaproate (KIC) (16). Finally, the BCKAs are transaminated into BCAAs, with Val from KIV, Leu from KIC, and Ile from KMV, by branched-chain amino acid aminotransaminases (BCATs) (17). In contrast, degradation of BCAAs mainly occurs in the cytosol via Ehrlich degradation of BCAAs (12). In fact, BCAAs can be converted back into BCKAs by BCATs. Then, BCKAs are further converted by α-keto acid decarboxylases (KDCs) and alcohol dehydrogenases (ADHs) into BCHAs: isobutanol from Val, isoamyl alcohol from Leu, and active amyl alcohol from Ile (Fig. 1).

The transamination step of the Ehrlich pathway was reported to be a rate-limiting step for this pathway (18, 19). This transamination reaction is catalyzed by BCATs (12). S. cerevisiae has two BCAT isoforms, mitochondria BCAT (mBCAT [Bat1], encoded by the *BAT1* gene) and cytosolic BCAT (cBCAT [Bat2], encoded by the *BAT2* gene). These two isozymes drive the bidirectional transamination reaction between the corresponding BCKAs and BCAAs, i.e., KIV/Val, KIC/Leu, and KMV/Ile, respectively, together with the cosubstrates (glutamate [Glu] and α-ketoglutarate [KG]), which act as the amino group donor and acceptor for Glu and KG, respectively. BCAT is one of the pyridoxal 5′-phosphate (PLP)-dependent aminotransaminase enzymes (20). Bat1 (393 amino acid residues, size 43.6 kDa) and Bat2 (376 amino acid residues, size 41.6 kDa) proteins share 77% identity in amino acid sequence. The difference in size results from the mitochondrial-targeting signal (MTS; amino acid residues 1 to 17 of Bat1), which is attached at the N terminus of Bat1 (17, 21). This MTS shuttles Bat1 into mitochondria. On the other hand, Bat2 is located in the cytosol (22). Several studies have investigated the effects of Bat1 and Bat2 (whether deleted or overexpressed) on BCHA production (18, 23–28).

Unlike yeast BCATs, several amino acid substitutions that alter enzymatic activity have been identified and studied in human BCATs, since these amino acid substitutions are related to human diseases (29–33). The best-studied engineering on the yeast BCATs is the substitution of the conserved catalytic lysine residue, Lys219, of Bat1 (corresponding to Lys202 of Bat2) to other amino acids, including Ala (K219A of Bat1, corresponding to K202A of Bat2), Arg (K219R, corresponding to K202R of Bat2), His (K202H of Bat2), and Met (K202M of Bat2) (34, 35). However, those amino acid substitutions led to diminished catalytic activity. Our recent study (26) also clarified that a single amino substitution at position 234 in Bat1, from Ala to Asp (A234D), affected BCHA production. Nonetheless, A234D also leads to the dysfunction of Bat1. Therefore, no amino acid substitutions that change the enzymatic activity or substrate specificity and that impact BCHA production have been reported.

Previously, enhancement of the availability of intracellular precursors or key intermediates, BCKAs, was one of the traditional strategies for increasing BCHA production (23, 25). However, the transamination step catalyzed by Bat1 and Bat2 is the rate-limiting step, as described previously. In the present study, we aimed to improve BCHA production in S. cerevisiae by altering enzymatic activity or catalytic preference of Bat1 and Bat2 through the rational design of BCATs. Other recent studies have attempted the rational design of targeted enzymes as a means of either creating or optimizing the specific features of biocatalysts, including substrate specificity, enantioselectivity, or thermostability (36–38). We here applied *in silico* computational analysis to screen for appropriate amino acid substitutions around the active site or substrate-binding site of Bat1 and Bat2 to change their activity or catalytic preference, leading to increased accumulation of BCKAs and production of BCHA.

## RESULTS

### *In silico* screening for amino acid substitutions on Bat2.

The transamination step of the Ehrlich degradation pathway is well-known as a rate-limiting step (18, 19). In S. cerevisiae, this transamination process is catalyzed by Bat1 or Bat2. These two isozymes have compatibility functions and impact BCHA production (18, 21, 25). However, many studies have suggested that Bat2 has a greater physiological role on BCHA production than Bat1 (6, 18, 19, 27, 39). Therefore, we performed an *in silico* analysis of Bat2.

**FIG 2:**
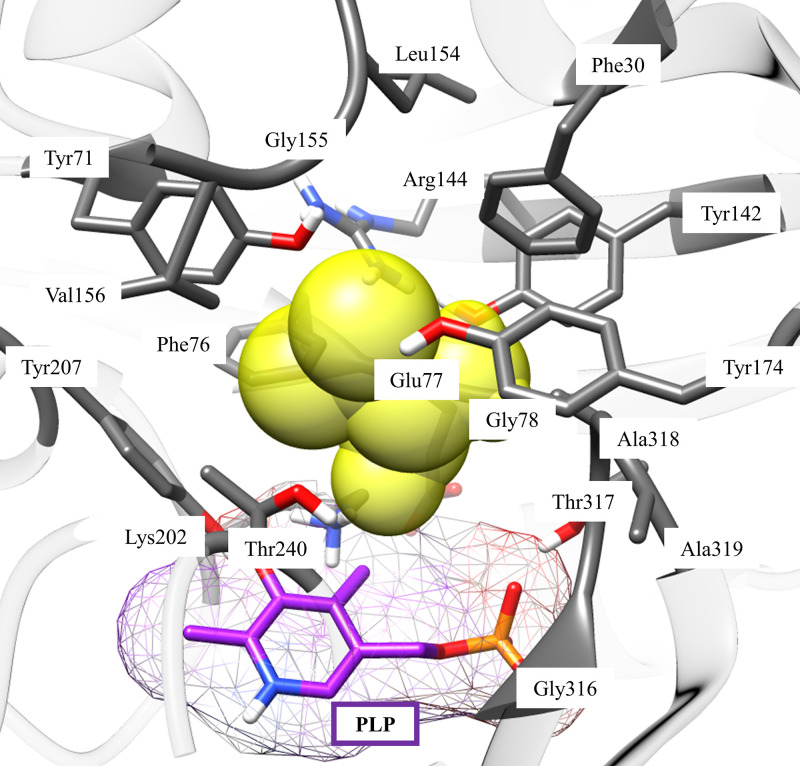
Amino acid residues interacting at the Bat2 active site. The yellow sphere represents substrates that bind to the Bat2 active site. Pyridoxal 5′-phosphate (PLP) is shown as a purple stick. Amino acid residues in the Bat2 active site that interact or surround substrates (5 Å) are shown as gray sticks.

To design the appropriate amino acid substitutions, we first modeled the homology structure of Bat2 with SWISS-MODEL (see Fig. S1 in the supplemental material) using the human cytosolic BCAT (SMTL ID 2abj.1 [chain A]) as a template (see Fig. S2). This step was necessary because the crystal structures of Bat1 and Bat2 have not yet been studied. Moreover, the active form of the yeast BCAT is similar to that of the human BCAT (as a dimer) (40, 41). Next, we performed a molecular docking simulation using AutoDock Vina to select the candidate residues of Bat2 (see Fig.S3 and S4 and Tables S2 and S3 in the supplemental material) and analyzed all the amino acid residues of Bat2 that interacted either directly or indirectly with substrates, BCKAs (KIV, KIC, and KMV) and BCAAs (Val, Leu, and Ile), within a distance of 5 Å from the substrates (Fig. 2). As a result, 18 amino acid residues of Bat2 were obtained: Phe30, Tyr71, Phe76, Glu77, Gly78, Tyr142, Arg144, Gly155, Val156, Tyr174, Lys202, Tyr207, Thr240, Gly316, Thr317, Ala318, and Ala319. Among them, six residues (Tyr71, Tyr142, Arg144, Lys202, Tyr207, and Thr317) were eliminated as candidates for engineering because they have been reported to interact with PLP or substrates directly via hydrogen bonds in BCATs from Escherichia coli and humans (42–44). Thus, a total of 12 residues (Phe30, Phe76, Glu77, Gly78, Leu154, Gly155, Val156, Tyr174, Thr240, Gly316, Ala318, and Ala319) that indirectly interacted with substrates (BCAAs and BCKAs) were designated for further *in silico* engineering.

We next performed a screening using CUPSAT, which can automatically change any of the original amino acid resides to any other amino acid residue. As a result, 82 substitutions on 12 designated residues in which protein stability was increased compared to the original amino acid residue, were selected: Phe30 (F30C), Phe76 (F76C), Glu77 (E77T, E77Q, E77K, E77D, and E77H), Gly78 (G78V, G78L, G78I, G78M, G78T, G78F, G78K, G78N, G78E, G78R, and G78H), Leu154 (L154G, L154M, L154T, L154Q, L154K, L154E, L154R, and L154H), Gly155 (G155A, G155P, G155T, G155F, G155Q, and G155C), Tyr174 (Y174G, Y174A, Y174V, Y174L, Y174I, Y174M, Y174P, Y174S, Y174T, Y174Q, Y174K, Y174N, Y174C, Y174E, Y174D, Y174R, and Y174H), Thr240 (T240P, T240W, T240S, T240F, T240Q, and T240C), Gly316 (G316L, G316I, G316W, G316S, G316T, G316Q, G316K, G316Y, G316N, G316E, G316D, G316R, and G316H), Ala318 (A318V, A318L, A318I, A318P, A318W, A318Q, A318Y, A318C, and A318H), and Ala319 (A319V, A319P, A319W, A319T, and A319R) (see Table S4 in the supplemental material). To screen for amino acid substitutions that might change the specific activity of variant BCATs, we further analyzed the effects of 82 amino acid substitutions on the substrate-binding affinities using mCSM-lig. Among them, 7 amino acid substitutions, Gly78 (G78N), Leu154 (L154Q), Tyr174 (Y174D), Gly316 (G316W and G316S), Ala318 (A318Q), and Ala319 (A319T), showed an increase in substrate-binding affinities compared with the original amino acid residue, especially when BCAA was the substrate (Table 1).

**TABLE 1:** Predicted effect of amino acid substitutions on substrate-binding affinities

Amino acid substitution	Change in substrate-binding affinity from the original amino acid residue^*a*^					
	KIV	KIC	KMV	Val	Leu	Ile
G78N	−0.059	0.006	−0.016	0.063	0.061	0.1
L154Q	0.031	0.106	−0.01	0.00	0.001	0.145
Y174D	0.366	0.383	0.401	0.473	0.552	0.48
G316W	−0.056	−0.011	0.016	0.037	0.248	0.081
G316S	0.638	0.716	0.749	0.824	1.034	0.888
A318Q	0.572	0.63	0.657	0.737	0.854	0.779
A319T	0.159	0.205	0.252	0.299	0.493	0.299

a The values were calculated as the logarithmic change of binding affinity from the original amino acid residue (calculated using mCSM-lig); positive and negative values indicate increased and decreased binding affinities, respectively.

*In silico* prediction results suggested that amino acid substitution from Gly to Ser at position 316 of Bat2 (G316S) increases the protein stability and substrate-binding affinities toward all BCAAs. Indeed, G316S was the best substitution candidate for further *in vivo* and *in vitro* investigation. Notably, other amino acid substitutions at position 316, particularly from Gly to Trp (G316W), were suggested to increase protein stability and to change substrate-binding affinities; this substitution increased binding affinities toward BCAAs but decreasing binding affinities toward BCKAs compared to an original residue. Therefore, these two amino acid substitutions of Bat2 (Bat2^G316S^ and Bat2^G316W^) were selected for further *in vivo* experiments.

### Effects of amino acid substitutions in BCATs on the growth phenotype.

**FIG 3:**
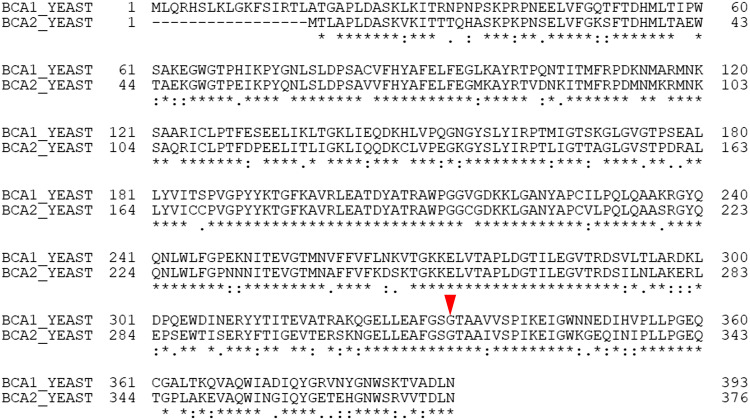
Amino acid sequence alignments of Bat1 and Bat2. The alignment was performed using the Clustal Omega program and the alignment tool in the Uniprot database. BCA1_YEAST and BCA2_YEAST indicate Bat1 and Bat2 of S. cerevisiae, respectively. The numbers are residue numbers. The red arrow indicates Gly333 in Bat1 and Gly316 in Bat2. Asterisks indicate fully conserved residue positions. Colons indicate conservation between groups of strongly similar properties (scoring > 0.5 in the Gonnet PAM 250 matrix). Periods indicate conservation between groups of weakly similar properties (scoring ≤0.5 in the Gonnet PAM 250 matrix). Dashes indicate the absence of corresponding amino acid residues at those positions.

The amino acid sequence alignments of Bat1 and Bat2 showed that Gly316 in Bat2 corresponds to Gly333 in Bat1 (Fig. 3). We then constructed both Bat1 and Bat2 variants that had the same amino acid substitutions, i.e., the Bat1 variants Bat1^G333S^ and Bat1^G333W^ and the Bat2 variants Bat2^G316S^ and Bat2^G316W^. The plasmids harboring the genes for wild-type Bat1 (Bat1^WT^) and its variants (Bat1^G333S^ and Bat1^G333W^) and for wild-type Bat2 (Bat2^WT^) and its variants (Bat2^G316S^ and Bat2^G316W^) were introduced into BY4741*bat1*Δ*bat2*Δ cells. The resultant transformants expressed only one BCAT isoform, to eliminate the redundancy effect of Bat1 and Bat2. The control strains used in this study were BY4741*bat1*Δ*bat2*Δ cells expressing Bat1^WT^ (strain Bat1-WT) and Bat2^WT^ (strain Bat2-WT), and the wild-type strain BY4741 with the empty vector (strain WT). Unfortunately, BY4741*bat1*Δ*bat2*Δ cells expressing Bat2^G316W^ (Bat2-G316W) were unable to grow. Thus, the other six transformant strains (WT, Bat1-WT, Bat1-G333S, Bat1-G333W, Bat2-WT, and Bat2-G316S) were used for further experiments.

**FIG 4:**
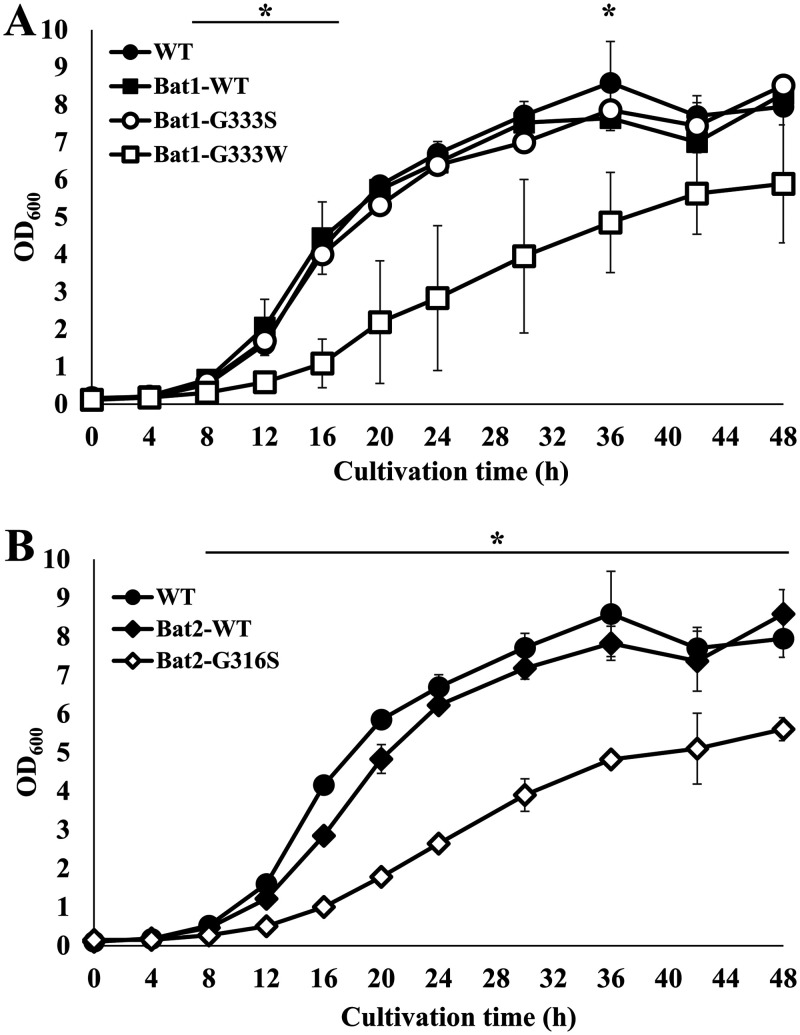
Effects on cell growth of amino acid substitutions in Bat1 and Bat2. (A) Growth phenotypes of BY4741*bat1*Δ*bat2*Δ cells expressing Bat1^WT^ (strain Bat1-WT), Bat1^G333S^ (strain Bat1-G333S), Bat1^G333W^ (strain Bat1-G333W), and the wild-type strain BY4741 with the empty vector (strain WT). (B) Growth phenotypes of BY4741*bat1*Δ*bat2*Δ cells expressing Bat2^WT^ (strain Bat2-WT), Bat2^G316S^ (strain Bat2-G316S), and the wild-type strain BY4741 with the empty vector (strain WT). Yeast cells were cultivated in SD medium. Each point represents the mean with standard deviations from three independent experiments. *, significant difference where *P *was <0.05 for Bat1-G333W and Bat2-G316S versus WT, verified by Student’s *t* test.

We next investigated the growth phenotypes of yeast cells expressing the different BCAT variants in minimal medium (Fig. 4). The growth of strain Bat1-G333S was almost the same as that of strains Bat1-WT and WT. In contrast, a significant growth delay was observed in strain Bat1-G333W compared to the other strains (Fig. 4A). In the case of Bat2, strain Bat2-G316S grew more slowly than strains Bat2-WT and WT (Fig. 4B).

### Effects of amino acid substitutions in BCATs on BCHA production.

**FIG 5:**
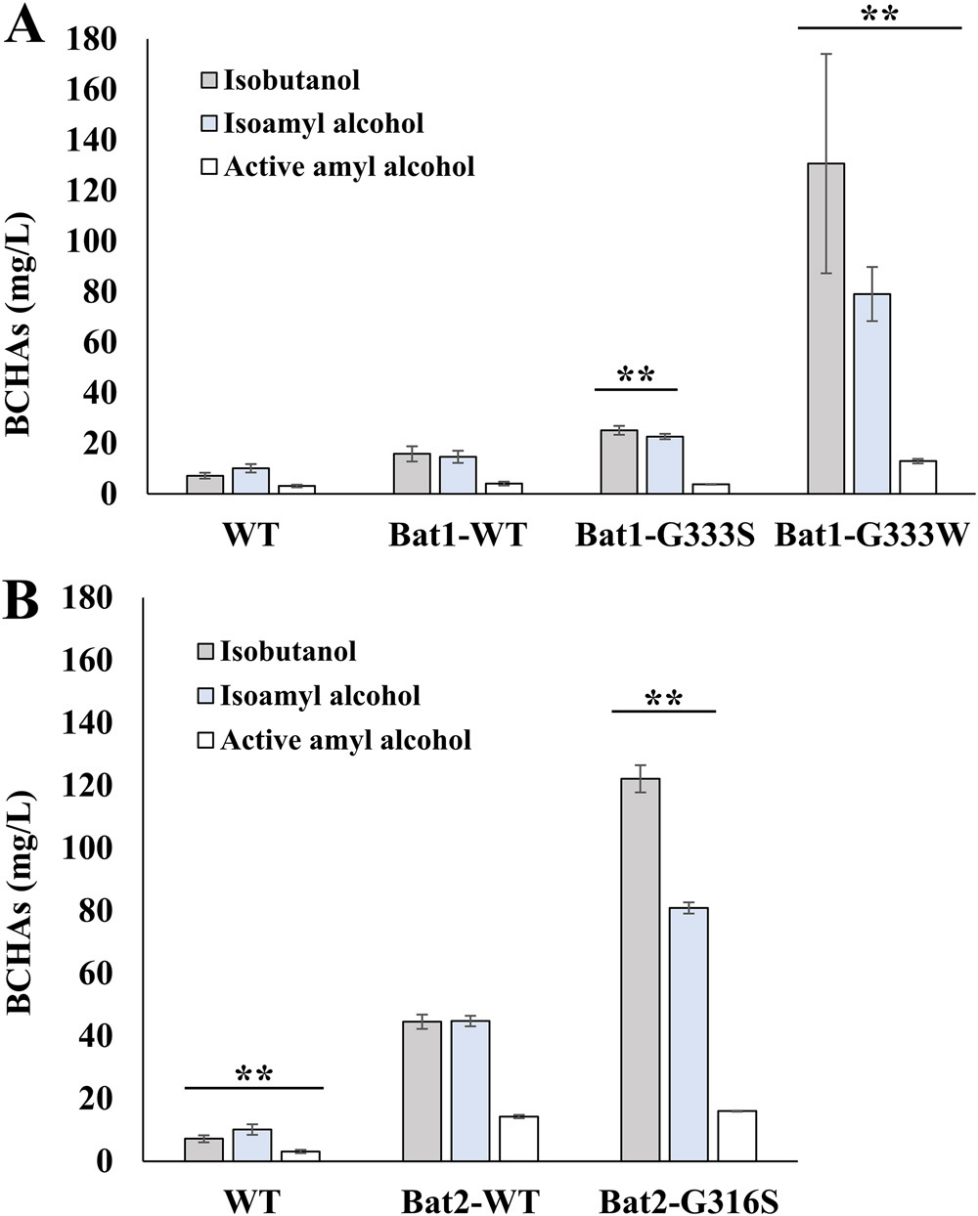
Effects of amino acid substitutions in Bat1 and Bat2 on fusel alcohol production. (A) BCHA contents of strains Bat1-WT, Bat1-G333S, Bat1-G333W, and WT. (B) BCHA contents of strains Bat2-WT, Bat2-G316S, and WT. Yeast cells were cultivated in SD medium for 3 days. The supernatants from each cultured broth were used to measure BCHA content by GC-MS. Each point represents the mean with standard deviations from three independent experiments. **, significant differences where *P *was <0.01 versus controls (Bat1-WT or Bat2-WT), verified by nonrepeated measured analysis of variance (ANOVA) followed by the Bonferroni correction.

Our previous study revealed that the growth defect of yeast cells lacking Bat1 is related to changes in the production of metabolites (26). Hence, we next determined the contents of BCHAs (isobutanol, isoamyl alcohol, and active amyl alcohol) in the fermentation broth after cultivation of yeast cells for 3 days (Fig. 5). We found that strains Bat1-G333S and Bat1-G333W exhibited higher amounts of isobutanol and isoamyl alcohol than strains Bat1-WT and WT. Surprisingly, all BCHA contents (isobutanol, isoamyl alcohol, and active amyl alcohol) of strain Bat1-G333W (131 ± 43, 79 ± 11, and 13 ± 1 ppm, respectively) were much higher than the corresponding contents in strains Bat1-WT (16 ± 3, 15 ± 2, and 4 ± 1 ppm, respectively) and WT (7 ± 1, 10 ± 2, and 3 ± 1 ppm, respectively) (Fig. 5A). Likewise, the BCHA contents (isobutanol, isoamyl alcohol, and active amyl alcohol) of strain Bat2-G316S (122 ± 4, 81 ± 2, and 16 ± 1 ppm, respectively) were also higher than those of strains Bat2-WT (45 ± 2, 45 ± 2, and 14 ± 1 ppm, respectively) and WT (7 ± 1, 10 ± 2, and 3 ± 1 ppm, respectively) (Fig. 5B). The levels of production of isobutanol, isoamyl alcohol, and active amyl alcohol in strains Bat1-G333W and Bat2-G316S were 18.7-fold and 17.4-fold, 7.9-fold and 8.1-fold, and 4.3-fold and 4.6-fold higher than those of strain WT, respectively.

### Effects of amino acid substitutions in BCATs on BCAA production.

**FIG 6:**
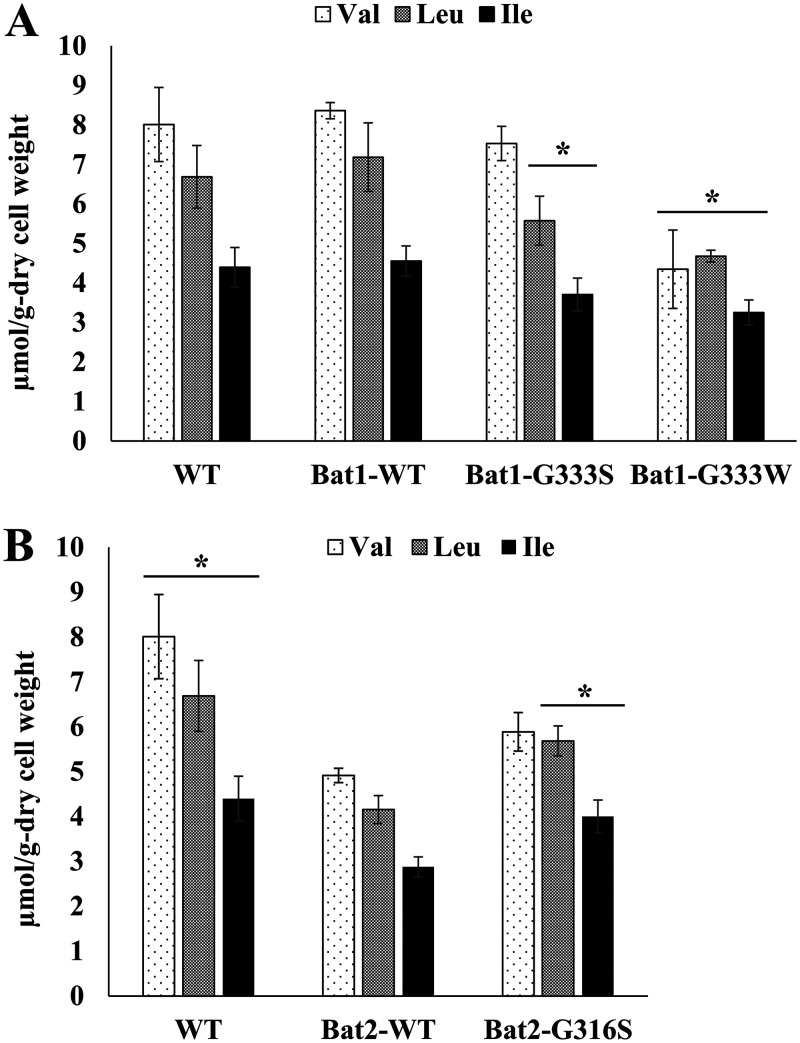
Effects of amino acid substitutions in Bat1 and Bat2 on BCAA production. (A) BCAA contents of strains Bat1-WT, Bat1-G333S, Bat1-G333W, and WT. (B) BCAA contents of strains Bat2-WT, Bat2-G316S, and WT. Yeast cells were cultured in SD medium for 2 days, and intracellular BCAA contents were analyzed by an amino acid analyzer. Each point represents the mean with standard deviations from three independent experiments. *, significant differences where *P *was <0.05 versus controls (Bat1-WT or Bat2-WT), verified by the nonrepeated measured ANOVA followed by the Bonferroni correction.

We next measured the intracellular contents of BCAAs, which are also important metabolites synthesized by BCATs (Fig. 6). Unlike in BCHAs, Leu and Ile contents in strain Bat1-G333S and all BCAA contents in strain Bat1-G333W were lower than those in strains Bat1-WT and WT (Fig. 6A). On the other hand, Leu and Ile contents in strain Bat2-G316S were higher than those of strain Bat2-WT, but lower than those of strain WT (Fig. 6B). Notably, in previous studies the BCAA contents of strain Bat2-WT were lower than those of strain WT (21, 26). These results suggest that the enzymatic properties of BCAT variants affect the contents of both extracellular BCHA and intracellular BCAA.

### Effects of amino acid substitutions in BCATs on enzymatic activity.

We next attempted to purify the recombinant BCATs from Escherichia coli BL21(DE3) cells. However, the wild-type Bat1 could not be purified using an Ni Sepharose 6 Fast Flow column (data not shown). A similar phenomenon was reported for the human BCAT (40). Hence, we removed the first 16 amino acid residues at the N terminus, to construct a truncated Bat1 named Bat1ΔN16^WT^. Those 16 amino acids are encoded as a mitochondrial-targeting signal (MTS) (21). Prohl et al. (41) also successfully purified a truncated Bat1 that lacked the amino acid residues 1 to 16, and this truncated Bat1 can be used to measure the enzymatic activity. Thus, we finally purified Bat1ΔN16^WT^, Bat2^WT^, Bat1ΔN16^G333S^, and Bat2^G316S^ from E. coli BL21(DE3) cells (see Fig. S5 in the supplemental material) and assayed the BCAT activities *in vitro* (Table 2).

**TABLE 2:** Kinetic parameters of wild-type and variant BCATs

Substrate	Enzyme	*K_m_* (mM)^*a*^	*k*_cat_ (s^−1^)^*a*^	*k*_cat_/*K_m_* (mM^−1^·s^−1^)
KIV	Bat1ΔN16^WT^	0.298 ± 0.100	9.55 ± 1.92	32.1
	Bat1ΔN16^G333S^	2.73 ± 3.24	2.05 ± 1.70	0.751
	Bat2^WT^	0.180 ± 0.0444	8.05 ± 1.02	44.7
	Bat2^G316S^	1.73 ± 0.826	1.79 ± 0.521	1.03
KIC	Bat1ΔN16^WT^	0.323 ± 0.101	15.4 ± 2.92	47.7
	Bat1ΔN16^G333S^	1.57 ± 0.505	4.95 ± 0.945	3.15
	Bat2^WT^	0.212 ± 0.0610	11.6 ± 1.82	54.7
	Bat2^G316S^	0.681 ± 0.189	5.42 ± 0.615	7.96
KMV	Bat1ΔN16^WT^	0.218 ± 0.111	4.73 ± 1.29	21.7
	Bat1ΔN16^G333S^	0.548 ± 0.455	1.46 ± 0.435	2.66
	Bat2^WT^	0.150 ± 0.0594	5.48 ± 1.04	36.5
	Bat2^G316S^	0.308 ± 0.134	2.01 ± 0.282	6.53
Val	Bat1ΔN16^WT^	0.454 ± 0.123	3.37 ± 0.37	7.42
	Bat1ΔN16^G333S^	7.70 ± 7.37	0.834 ± 0.319	0.108
	Bat2^WT^	0.511 ± 0.111	2.71 ± 0.23	5.30
	Bat2^G316S^	13.3 ± 3.44	1.40 ± 0.17	0.106
Leu	Bat1ΔN16^WT^	0.285 ± 0.0679	3.51 ± 0.31	12.3
	Bat1ΔN16^G333S^	3.08 ± 1.42	0.701 ± 0.102	0.228
	Bat2^WT^	0.189 ± 0.0600	2.30 ± 0.24	12.2
	Bat2^G316S^	3.89 ± 1.06	1.61 ± 0.15	0.414
Ile	Bat1ΔN16^WT^	0.220 ± 0.102	3.74 ± 0.59	17.0
	Bat1ΔN16^G333S^	4.23 ± 5.39	0.769 ± 0.312	0.182
	Bat2^WT^	0.119 ± 0.0389	2.61 ± 0.24	21.9
	Bat2^G316S^	3.40 ± 1.14	1.80 ± 0.20	0.529

a The values are the means ± standard deviations of results from three independent experiments.

When BCKAs (KIV, KIC, and KMV) were used as substrates for BCAA synthesis (i.e., for monitoring the forward reaction of BCAT), Bat1ΔN16^G333S^ exhibited higher apparent *K_m_* values than did Bat1ΔN16^WT^. Bat2^G316S^ also showed apparent *K_m_* values toward BCKAs that were higher than the apparent *K_m_* values of Bat2^WT^. Also, the apparent *k*_cat_ values of Bat1ΔN16^G333S^ and Bat2^G316S^ were lower than those of Bat1ΔN16^WT^ and Bat2^WT^. Therefore, the apparent *k*_cat_/*K_m_* values of Bat1ΔN16^G333S^ and Bat2^G316S^ were significantly lower than those of Bat1ΔN16^WT^ and Bat2^WT^. Using BCAAs as a substrate for monitoring the reverse reaction of BCAT, we found that Bat1ΔN16^G333S^ and Bat2^G316S^ also displayed a similar trend in their forward reactions, which reduced the catalytic properties compared with those of Bat1ΔN16^WT^ and Bat2^WT^, respectively. The apparent *K_m_* values of Bat1ΔN16^G333S^ and Bat2^G316S^ were significantly higher than those of Bat1ΔN16^WT^ and Bat2^WT^. Similarly, both Bat1ΔN16^G333S^ and Bat2^G316S^ exhibited apparent *k*_cat_ values that were significantly lower than those of Bat1ΔN16^WT^ and Bat2^WT^. The significantly higher *K_m_* and lower *k*_cat_ values of BCAT variants eventually led to a major reduction in apparent *k*_cat_/*K_m_* values of Bat1ΔN16^G333S^ and Bat2^G316S^ compared to the corresponding values for Bat1ΔN16^WT^ and Bat2^WT^. In conclusion, all of the kinetic parameters of the BCAT variants (Bat1ΔN16^G333S^ and Bat2^G316S^) showed an increase in the *K_m_* values with a decrease in the *k*_cat_ and *k*_cat_/*K_m_* values relative to the corresponding wild-type enzymes (Bat1ΔN16^WT^ and Bat2^WT^), indicating that the catalytic activities of the BCAT variants were reduced.

## DISCUSSION

Here, we engineered Bat1 and Bat2 by *in silico* computational analysis and then investigated the effects of the Bat1 and Bat2 variants on the cell growth and production of metabolites (BCAAs and BCHAs). Finally, we examined the effects of amino acid substitutions on catalytic properties using the recombinant enzymes. The parental yeast strain BY4741*bat1*Δ*bat2*Δ has an auxotrophic phenotype in culture media without BCAAs (17, 41). Thus, the BCAAs produced by a functional Bat1 or Bat2 are crucial for the cell growth of those transformants in minimal media without BCAA supplementation. In this study, the phenotypes of BY4741*bat1*Δ*bat2*Δ cells expressing Bat1^WT^ or Bat2^WT^ were consistent with those in the previous studies (21, 26). Hence, we concluded that all BCATs (Bat1^WT^, Bat1^G333S^, Bat1^G333W^, Bat2^WT^, and Bat2^G316S^) are functional.

Interestingly, yeast cells expressing Bat1^G333S^, Bat1^G333W^, and Bat2^G316S^ produced higher levels of isobutanol and isoamyl alcohol than the cells expressing Bat1^WT^ and Bat2^WT^ (Fig. 5). The BCHA productivity of yeast cells expressing BCAT variants was correlated with the variants’ enzymatic activities (Table 2). In fact, the kinetic parameters of Bat1^G333S^ and Bat2^G316S^ showed lower apparent *k*_cat_ and higher apparent *K_m_* values than those of Bat1^WT^ and Bat2^WT^, respectively. Therefore, the apparent *k*_cat_/*K_m_* values of the BCAT variants were lower than those of Bat1^WT^ and Bat2^WT^, which was particularly true of the *k*_cat_/*K_m_* values toward BCKAs (synthesis of BCAAs), in association with a decrease in BCAA contents compared to the WT strain (Fig. 6). Hence, the high-level production of BCHAs by BCAT variants was probably due to a decrease in the catalytic activities of the BCAT variants. The enzymatic analysis also indicated that Bat1^WT^ and Bat2^WT^ prefer to synthesize BCAAs (using BCKAs as the substrates) under certain conditions. However, the *k*_cat_/*K_m_* values toward BCKAs of BCAT variants (Bat1^G333S^ and Bat2^G316S^) were significantly lower than those for Bat1^WT^ and Bat2^WT^, which would promote the efficiency of BCHA production. In other words, the low catalytic activity of BCAT variants directly decreased the rate of the reaction competing with BCHA production, namely, BCAA biosynthesis. Our recent study suggested that in yeast cells lacking Bat1, elevated levels of BCKAs, which are produced in mitochondria and then flow from the mitochondria to cytosol, are involved in an increase in BCHA levels (26). Moreover, BCAAs cause feedback inhibition of key enzymes involved in the biosynthetic pathway (15, 16), which would also affect the BCKA pool and, consequently, BCHA biosynthesis. The decreased catalytic activities of BCAT variants may reduce the feedback inhibition by BCAA, as the BCAA contents in strains Bat1-G333S and Bat1-G333W were lower than those in strain WT (Fig. 6).

**FIG 7:**
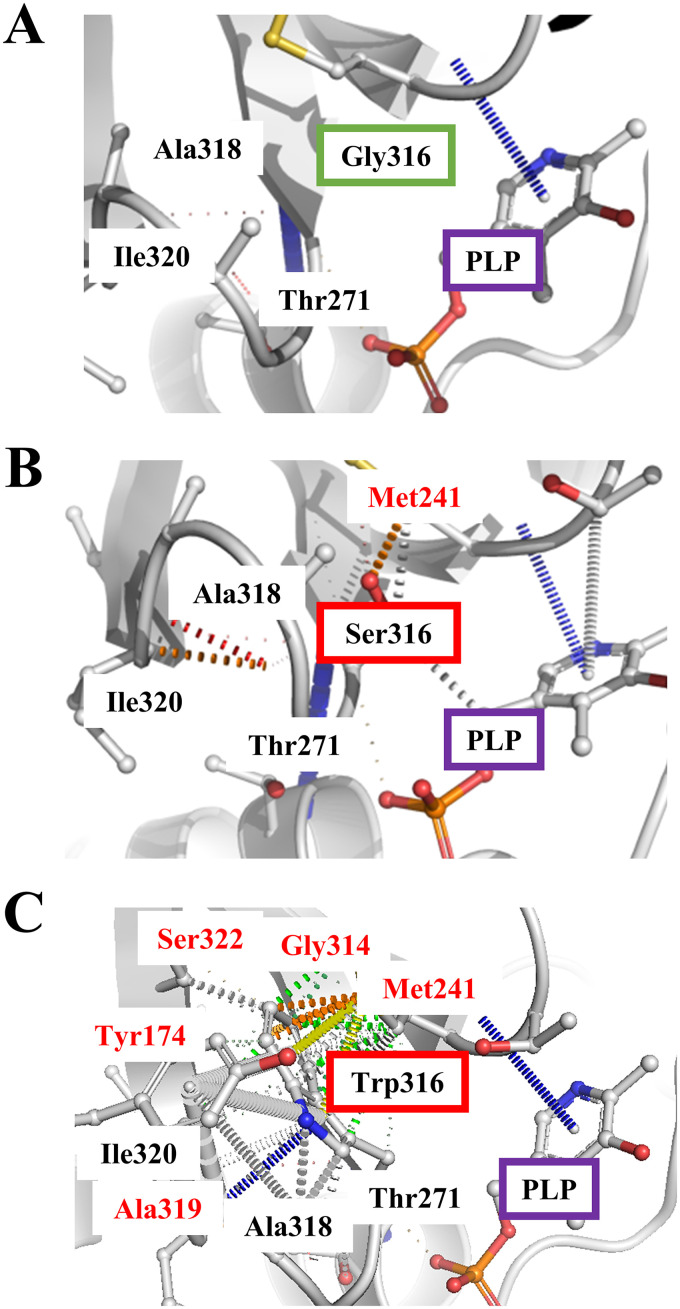
Schematic interatomic interactions between amino acid residues at position 316 in Bat2^WT^ (A), Bat2^G316S^ (B), and Bat2^G316W^ (C). Gly316, Ser316, and Trp316 are shown in green, red, and red boxes, respectively. The amino acid residues that interact with Ser316 and Trp316 but do not interact with Gly316 are shown with red fonts.

**FIG 8:**
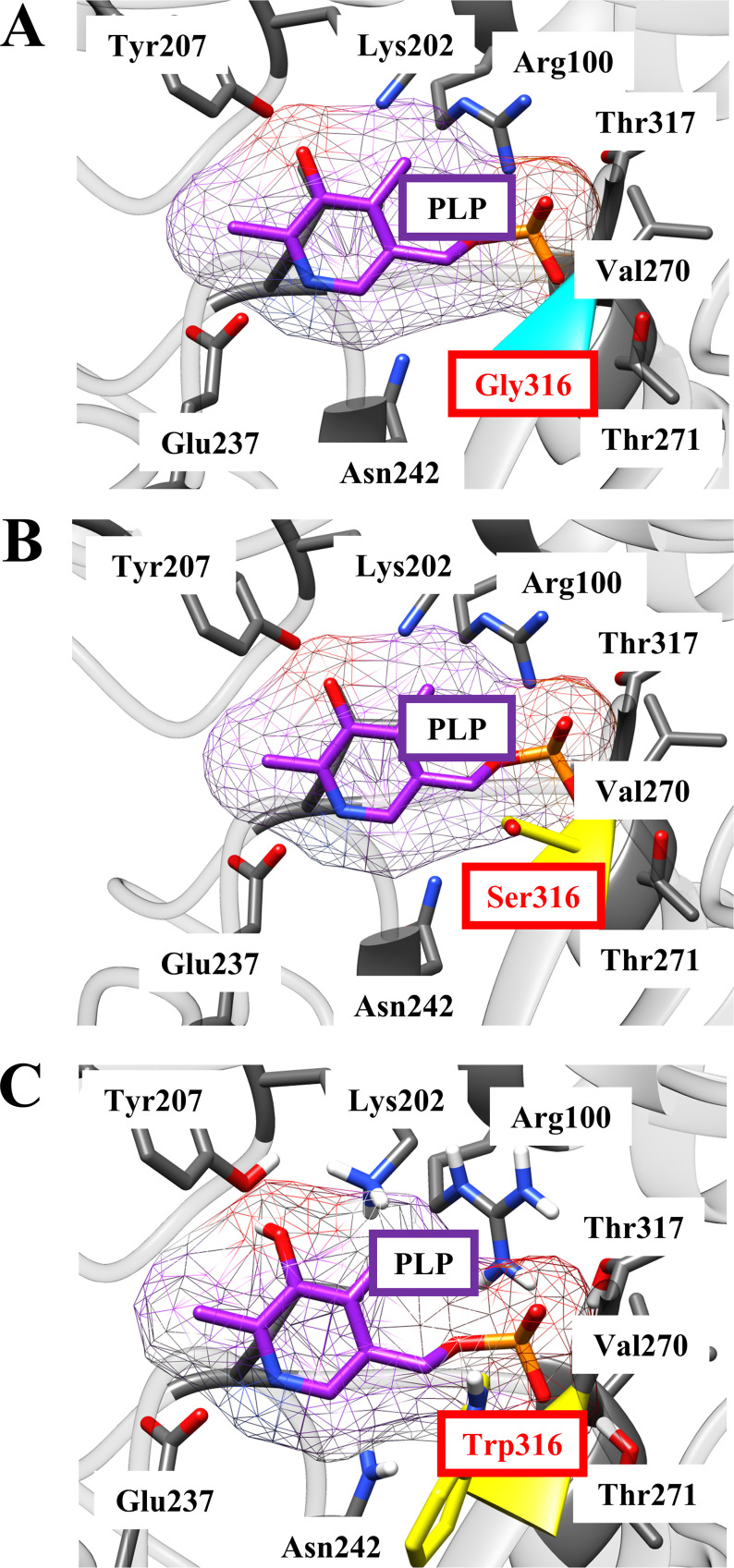
Schematic active center of Bat2^WT^ (A), Bat2^G316S^ (B), and Bat2^G316W^ (C). Gly316 in Bat2^WT^ is marked in cyan. Ser316 and Trp316 in Bat2^G316S^ and Bat2^G316W^, respectively, are marked in yellow color. The distance between Ser316 or Trp316 and the phosphate group of PLP (orange-red end) is ≈4.5 Å (measured with UCSF Chimera).

The results shown in Table 2 suggest that the abilities of the BCAT variants to bind with substrates and to convert substrates into products of variant enzymes were lower than those of the wild-type enzyme. By *in silico* comparative analysis of the structures of Bat2^G316S^ and Bat2^WT^ (Fig. 7A and B), Ser316 was found to display an additional interaction with Met241 that did not exist in Bat2^WT^. In the human BCAT, Asn242 is known to directly interact with a pyridoxal 5′-phosphate (PLP) cofactor (see Fig. S2 in the supplemental material) (43). Met241 is located near Asn242. Thus, an additional interaction between Ser316 and Met241 would impact the catalytic engines of Bat2^G316S^. Moreover, the amino acid residue at position 316 of Bat2 is located in close proximity to the phosphate group of PLP (within 4.5 Å) (Fig. 8A and B), which is the catalytic center where transamination reactions occur (45). The side chain of Ser, which is larger and bulkier than that of Gly (46), easily blocks or affects substrate binding and interaction with the phosphate group of PLP. Hence, a Gly-to-Ser substitution at position 316 in Bat2 may affect the enzymatic properties. It is noteworthy that Ser316 and Met241 in Bat2 were conserved in Bat1 (Ser333 and Met258) (Fig. 3). In fact, the kinetic parameters were similar between Bat1^G333S^ and Bat2^G316S^, indicating that the Gly-to-Ser substitution decreases the catalytic activity of BCAT. However, the phenotypes of cell growth and BCHA production in strain Bat2-G316S were quite distinct from those of strain Bat1-G333S (Fig. 4 and 5). These differences could be explained by the differences in localization, stability, expression, and transcriptional regulation between Bat1 and Bat2 (22, 47, 48). In fact, yeast cells expressing only Bat2 in the cytosol (*bat1*Δ cells) basically exhibited a growth defect with an increase in BCHA content compared to yeast cells expressing only Bat1 in mitochondria (*bat2*Δ cells) (21, 25, 26) (Fig. 5). Accordingly, Ser substitution at position 316 on Bat2 has a greater impact on the phenotypes of cell growth and BCHA production than Ser substitution at position 333 on Bat1.

Interestingly, strain Bat1-G333W exhibited a major increase in BCHA levels but also a decrease in BCAA content compared to the other strains (Fig. 5A and 6A). The *in silico* investigation of Bat2 revealed that Trp substitution at position 316 resulted in additional intramolecular interactions with many more residues (Tyr174, Gly314, Ala319, and Ser322) compared to Ser substitution at position 316 (Fig. 7B and C). It is worth noting that these residues were located within the area of substrate binding (within 5 Å) and indirectly interacted with substrates (see Tables S2 and S3 in the supplemental material). Importantly, these residues (Tyr174, Gly314, Ala319, and Ser322 of Bat2) are fully conserved in Bat1 (Tyr191, Gly331, Ala336, and Ser339 of Bat1) (Fig. 3). Indeed, Trp substitution in Bat1 has more impact on enzymatic properties than Ser substitution. This Trp substitution leads to the lower catalytic activity of Bat1^G333W^ compared to Bat1^G333S^, and it consequently has a higher impact on cell growth and BCHA production. Moreover, while a lack of Bat1 is a well-known and effective approach to overproduce BCHA (25, 26, 49), it is intriguing that the BCHA production of strain Bat1-G333W was much greater than in those of strains WT and Bat1-WT. Another important phenotype of strains Bat1-G333W and Bat2-G316S is the growth defect when cultured in minimal medium (Fig. 4), which was considered to be related to metabolite production. Actually, the productivities of BCAAs in these strains were significantly lower than in the wild-type strain (Table 2). Such insufficient levels of BCAAs might affect cell growth of the BCAT variants, consistent with the lower BCAA contents of Bat1-G333W and Bat2-G316S compared to the wild-type strain (Fig. 6A and B).

In this study, the BCAT variants (Bat1ΔN16^G333S^ and Bat2^G316S^) showed a decrease in catalytic activity compared to the wild-type Bat1 and Bat2 (Bat1ΔN16^WT^ and Bat2^WT^) (Table 2), even though the *in silico* analysis indicated that the Bat2 variants had greater binding affinities toward the substrates (Table 1). The *in silico* simulations (molecular docking and mCSM-lig) that were mainly used in this study are static and fairly rough determinants of the properties of BCAT variants based on binding affinity with the substrate. These methods are currently the most effective to clarify or predict their biological performance of enzyme variants (50). However, the catalytic activity of an enzyme is highly dynamic; conformational fluctuation or movement of amino acids around the active site and vibration of the backbone interrelate to yield the characteristic catalytic function (51–53). A recent study clarified a catalytic mechanism of histidine decarboxylase, one of the PLP-dependent enzymes, based on molecular dynamics simulation and hybrid quantum mechanics and molecular mechanics (QM/MM) studies (54). Also, the unbinding of ligands (substrates or products of enzyme) has been reported to have a positive effect on enzyme turnover, which can improve the biocatalyst properties of an enzyme (55). Hence, complementing static simulations with dynamic simulations, including molecular dynamic simulation, QM/MM studies, and the unbinding kinetics of ligands, would deepen our understanding of the enzyme dynamics and could eventually yield BCAT variants with improved catalytic activities for specific biotechnological applications.

In terms of the efficiency of BCHA production, strains Bat1-G333W and Bat2-G316S produced approximately 20 times more isobutanol than the wild-type strain in synthetic dextrose (SD) minimal medium with 2% glucose (Fig. 5). A previous study reported an ≈13-fold increase in isobutanol production in the engineered strain BY4741 with deletion of *BAT1* and overexpressing *ILV*, which is involved in BCKA biosynthesis, when cultured in synthetic complete (SC) medium with 10% glucose (25). Moreover, the isoamyl alcohol and active amyl alcohol contents were also remarkably increased in strains Bat1-G333W and Bat2-G316S (Fig. 5). In the present study, we propose a novel approach for overproducing BCHAs by using engineered BCATs with altered catalytic activities. Using the Bat1 and Bat2 variants, further studies can be performed to enhance BCHA productivity by combination with the previous successful approaches: engineering of other enzymes, mitochondrial compartmentalization of the BCHA biosynthetic pathways, and increase of glucose content or nutrients in the culture medium (25, 56, 57).

## MATERIALS AND METHODS

### *In silico* design of Bat2 variants.

The homology structure of Bat2 with PLP was modeled from the SWISS-MODEL (58) using human cytosolic BCAT (hBCATc; SMTL ID 2abj.1; chain A) as a template. Structural validation was validated based on homology structure quality by SWISS-MODEL quality estimation (QSQE, GMQE, QMEAN) connecting with SWISS-MODEL web server and PROCHECK analysis (Ramachandran plots and G-factors) (59), connecting with the PDBsum database (60). The structures of Bat2 variants were constructed using the Rotamers tool, connecting with UCSF Chimera (61), using the Dunbrack (2010) Rotamer library (62). Structural minimization of the wild-type and variants of Bat2 was performed in UCSF Chimera to remove atomic clashes and contacts using the 1,000 steepest descent steps (step size, 0.02 Å). The net charge of PLP (−2) was computed using ANTECHAMBER (63). The interatomic interaction between Bat2 and its variant structures was analyzed and visualized with the Arpeggio web server (64).

Density functional theory-optimized structures of the BCAAs (Val, Leu, and Ile) and BCKAs (KIV, KIC, and KMV) were obtained using the Gaussian 09 program package (65) at the B3LYP/6-311+G(d,p) level of theory (66–68). All the B3LYP/6-311+G(d,p)-optimized structures were converted to the format of the Protein Data Bank (PDB) for molecular docking. The most robust binding was considered the most stable configuration based on each docking simulation’s binding affinity or energy. The docking simulations were performed using the AutoDock Vina v.1.1.2 (69). Docking simulations and analyses were performed using the ADT/PMV/Viewer v.1.5.6 software (70). The interaction configurations between the Bat2 and variant proteins with all substrates were analyzed and visualized using the LigPlot+ v. 2.2.4 software via the academic free license (71). The catalytic center of Bat2 and its variants were visualized using UCSF Chimera.

Preliminary screening of amino acid substitutions for Bat2 was performed using the CUPSAT web server (72). The mutations, which affect protein stability, were quantified as the change in folding free energy (ΔΔ*G*). CUPSAT estimates ΔΔ*G* upon mutation by using mean force atom pair and torsion angle potential. The amino acid substitutions that stabilize the folding stability of the protein structure were selected for further analysis. An effect of mutations on protein-small molecule affinity was further investigated by using the mCSM-lig web-server (73). mCSM-lig can predict the change in binding affinity upon mutation compared to that with the original amino acid residue using a graph-based signature. Hence, the amino acid substitutions that stabilized substrate-binding affinities (i.e., increased substrate-binding affinities than with the original amino acid residue) were selected.

### Strains and media.

The yeast S. cerevisiae strain BY4741*bat1*Δ*bat2*Δ (BY4741Δ*bat1*::*kanMX6*Δ*bat2*::*hphNT1*), constructed as for the previous study (21), was used as a host strain for the construction of transformants. S. cerevisiae strain BY4741 (*MAT*α *his3*Δ*1 leu2*Δ*0 met15*Δ*0 ura3*Δ*0*) was also used as an original wild-type strain. An Escherichia coli DH5α strain [F^−^ ϕ80d*lacZ*ΔM15 Δ(*lacZYA-argF*)*U169 deoR recA1 endA1 hdR17* (r_K_^−^ m_K_^+^) *phoA supE44* λ^−^ *thi-1 gyrA96 relA1*] was used as a host for the construction and extraction of plasmids. Yeast strains were cultured in a nutrient-rich yeast extract-peptone-dextrose (YPD) medium {10 g/L yeast extract (Becton Dickinson [BD], Franklin Lakes, NJ), 20 g/L peptone (BD), and 20 g/L glucose} or a synthetic dextrose (SD) minimal medium (1.7 g/L yeast nitrogen base without amino acid [BD], 5 g/L ammonium sulfate, and 20 g/L glucose at pH 6.0). The antibiotics Geneticin (150 μg/mL) and hygromycin B (50 μg/mL) were appropriately supplemented to maintain the gene-disrupted status. An E. coli DH5α strain was cultivated in Luria-Bertani (LB) complete medium (5 g/L yeast extract, 10 g/L tryptone [BD], and 10 g/L NaCl). Cultures were supplemented with ampicillin (100 μg/mL) to maintain the transformant status. For the bacterial cells harboring pDONR221, 50 μg/mL of kanamycin was added to LB medium. Otherwise, the bacterial cells harboring pET53 were cultured in LB with an addition of 100 μg/mL ampicillin. In the case of BCAT proteins expressed in E. coli BL21(DE3) cells (F^–^ *ompT hsdS*(r_B_^–^m_B_^–^) *gal dcm* λ(DE3) (*lacI lacUV5-T7 gene1 ind1 sam7 nin5*), bacterial cells were cultured in M9 (4 g/L glucose, 246 mg/L MgSO_4_ · 7H_2_O, 6 g/L Na_2_HPO_4_, 3 g/L KH_2_PO_4_, 0.5 g/L NaCl, and 1 g/L NH_4_Cl) plus 20 g/L casamino acid (M9CA) with supplementation of ampicillin (100 μg/mL). If necessary, all media were solidified by adding 2% agar.

### Construction of expression plasmids and transformants.

BCAT proteins were expressed in S. cerevisiae BY4741*bat1*Δ*bat2*Δ using pRS416-*BAT1* and pRS416-*BAT2*, which were used in the previous study (21). Site-directed mutagenesis was performed using pRS416-*BAT1* and pRS416-*BAT2* as templates. The following primers were used to introduce mutations in the *BAT1* gene: from Gly333 to Ser333, G333S_F (5′-GCC TTC GGT TCT AGT GCT GCT GTC-3′) and G333S_R (5′-GAC AGC AGC AGT ACT AGA ACC GAA GGC-3′), and from Gly333 to Trp333, G333W_F (5′-GCC TTC GGT TCT TGG ACT GCT GCT GTC-3′) and G333W_R (5′-GAC AGC AGC AGT CCA AGA ACC GAA GGC-3′). The following primers were used to introduce mutations in the *BAT2* gene: from Gly316 to Ser316, G316S_F (5′- GCC TTT GGT TCT AGT ACT GCT GCG ATT-3′) and G316S_R (5′-AAT CGC AGC AGT ACT AGA ACC AAA GGC-3′), and from Gly316 to Trp316, G316W_F (5′-GCC TTT GGT TCT TGG ACT GCT GCG ATT-3′) and G316W_R (5′- AAT CGC AGC AGT CCA AGA ACC AAA GGC-3′). The double-underlined nucleotides reveal the corresponding amino acid substitution in Bat1 and Bat2. The subsequent PCR products were cut with DpnI and transformed into E. coli DH5α cells (74). After obtaining PCR products and designed plasmids, DNA sequences of the products were verified by using a DNA sequencer (ABI PRISM 3130 Genetic Analyzer; Applied Biosystems, Waltham, MA, USA). Corresponding Bat1 variants (Bat1^G333S^ and Bat1^G333W^) and Bat2 variants (Bat2^G316S^ and Bat2^G316W^) in pRS416 series were cotransformed with plasmid pRS415-Cg*HIS3MET15* (21) using the high-efficiency transformation method (75) to complement the auxotrophic phenotype of the BY4741 strain. SD agar plates were used to select the transformant colonies for corresponding experiments.

For expression of BCATs using E. coli BL21(DE3) cells, the *BAT1* gene was amplified from genomic DNA of S. cerevisiae BY4741 using the following primers: F-*BAT1*ΔN16-attB1 (5′ GGG GAC AAG TTT GTA CAA AAA AGC AGG CTT ACT CGC TAC TGG TGC CCC ATT-3′) and R-*BAT1*-attB2 (5′-GGG GAC CAC TTT GTA CAA GAA AGC TGG GTT GTT CAA GTC GGC AAC AGT TT-3′). The resultant PCR fragments of *BAT1* were introduced into the pDONR221 vector (Thermo Scientific, Waltham, MA, USA) using BP clonase II (Thermo Scientific), resulting in pDONR221-*BAT1*ΔN16. Site-directed mutagenesis was performed using pDONR221-*BAT1*ΔN16 as a template and using the same primers as for pRS416-*BAT1* variants construction, resulting in pDONR221-*BAT1*ΔN16^G333S^. For the construction of pDONR221-*BAT2* and pDONR221-*BAT2* variants, both the wild-type and mutant *BAT2* genes were directly amplified from pRS416-*BAT2* and pRS416-*BAT2* variants by using the following primers: F-*BAT2*-attB1 w/o SD (5′- GGG GAC AAG TTT GTA CAA AAA AGC AGG CTT AAT GAC CTT GGC ACC CCT AGA-3′) and R-BAT2-attB2 w/o SC (5′-GGG GAC CAC TTT GTA CAA GAA AGC TGG GTT GTT CAA ATC AGT AAC AAC CCT-3′). The following resultant PCR fragments of the *BAT2* series were also introduced into the pDONR221 vector by using BP clonase II, resulting in pDONR221-*BAT2* and pDONR221-*BAT2*^G316S^. DNA sequences of the *BAT1* series and *BAT2* series in pDONR221 were confirmed and transferred into a pET53-dest expression vector (Thermo Scientific) using LR clonase II (Thermo Scientific). Finally, the corresponding E. coli expression vectors with BCATs, pET53-*BAT1*ΔN16, pET53-*BAT1*ΔN16^G333S^, pET53-*BAT2*, and pET53-*BAT2*^G316S^ were obtained.

### Measurement of cell growth.

Yeast cells were precultured in 5 mL of SD medium at 30°C for 18 h. Then, the cell suspensions were transferred to 50 mL of SD medium with an initial optical density at 600 nm (OD_600_) of 0.1. The OD_600_ was measured every 4 h until the culture time reached 24 h. After 24 h, the OD_600_ was measured every 6 h until the culture time reached 48 h in order to trigger the growth phenotype.

### Measurement of BCAT metabolites.

Yeast cells were precultured in 5 mL of SD medium at 30°C for 18 h. Then, the cell suspensions were transferred to 50 mL of SD medium with an initial OD_600_ of 0.1. After 48 h of incubation, yeast cells were collected and adjusted an OD_600_ of 10. Amino acid contents were quantified using the same method as the previous study (26).

For the quantification of fusel alcohols content, the supernatants obtained from yeast cells cultured in SD medium with shaking for 3 days were collected. Gas chromatography-mass spectrometry (GC-MS) was also used to quantify fusel alcohol content, with a similar method as in the previous study (26).

### Expression and purification of the recombinant BCATs.

N-terminal His-tagged recombinant BCATs were expressed using E. coli BL21(DE3) cells harboring pET53-*BAT1*ΔN16, pET53-*BAT1*ΔN16^G333S^, pET53-*BAT2*, and pET53-*BAT2*^G316S^. DE3 cells were precultured in LB medium containing ampicillin at 37°C for 18 h. Then, the cell suspensions were transferred to 50 mL M9CA with an initial OD_600_ of 0.05. The cells were cultured at 37°C until the OD_600_ reached 0.6 to 0.8. Isopropyl β-D-1-thiogalactopyranoside (IPTG) at 0.25 mM and 0.5 mM concentrations for the series of Bat1 and Bat2, respectively, was added to induce protein expression. After cultivation at 18°C for 20 h, the cells were harvested by centrifugation and resuspended in 5 mL of buffer A (50 mM Tris-HCl [pH 8] and 300 mM NaCl]. The cell suspensions were homogenized and insoluble fractions were removed by centrifugation under cooling. The supernatants were loaded onto the nickel affinity column (Ni Sepharose 6 Fast Flow; GE Healthcare Life Sciences). After washing the column with buffer A containing 20 mM and 40 mM imidazole (for Bat1 and Bat2, respectively), buffer A with 500 mM imidazole was applied to the column to elute the recombinant BCATs. Dialysis was performed to remove imidazole together with exchanging the buffer from Tris-HCl to potassium phosphate and addition of dithiothreitol (DTT) to maintain and maximize the recombinant BCAT activities (40) using buffer B which contained 50 mM potassium phosphate buffer (pH 8.0), 300 mM NaCl, and 1 mM DTT.

### Assay of BCAT activity.

The recombinant BCAT activities were measured from triggering the production or extermination of NADH by coupling the transamination reaction with NAD-dependent glutamate dehydrogenase (GTD-211; Toyobo, Osaka, Japan) as described for previous studies with some modification (41, 47, 76) (see Fig. S2 in the supplemental material). The NADH level was monitored by measuring the absorbance at 340 nm using a DU-800 spectrophotometer (Beckman Coulter, Brea, CA). All reaction mixtures were maintained at 30°C for 15 min, with pre-equilibration of the reaction mixture for 2 min at 30°C. The forward reaction of BCAT was defined as conversion of BCKAs (KIV, KIC, and KMV) to BCAAs (Val, Leu, and Ile) as monitored by NADH production. In contrast, the reverse reaction of BCAT was defined as a conversion of BCAAs (Val, Leu, and Ile) to BCKAs (KIV, KIC, and KMV), which was monitored by the extermination of NADH. Amino acid group donor (Glu) and receptor (KG) was added to forward (Glu was added) and reverse (KG was added) reaction mixtures with the appropriate concentration.

Steady-state kinetics of the recombinant BCATs were assayed for both forward and reverse reactions of BCAT. For determining steady-state kinetics for the forward reaction assay, the concentration of Glu was kept at 100 mM for both wild-type and variant recombinant BCATs. Meanwhile, the concentration of BCKAs was varied (0.075 to 5 mM and 0.15 to 20 mM, respectively for the wild type [Bat1ΔN16^WT^ and Bat2^WT^] and variant [Bat1ΔN16^G333S^ and Bat2^G316S^] BCATs. The other mixture components for determination of steady-state kinetics for the forward reaction assay contained: 200 mM potassium phosphate buffer (pH 8.0), 0.25 mM NADH, 100 mM NH_4_Cl, 50 μM PLP, 100 mM Glu, 12 U of glutamate dehydrogenase (GDH), and 2 μg of purified recombinant BCATs. Each BCKA at a certain concentration was added to initiate the forward reaction. Otherwise, for determining the steady-state kinetics for the reverse reaction assay, the concentration of KG was kept at 2 mM for both wild-type and variant recombinant BCATs. The concentration of BCAAs was varied (0.25 to 50 mM and 5 to 150 mM for wild-type [Bat1ΔN16^WT^ and Bat2^WT^] and variant [Bat1ΔN16^G333S^ and Bat2^G316S^] BCATs, respectively). Then, 2 mM KG was added to initiate the reverse reaction. The other mixture components in determining the steady-state kinetics for the reverse reaction contained 200 mM potassium phosphate buffer (pH 8.5), 5 mM NAD^+^, 50 μM PLP, 24 U of GDH, and 2 μg of purified recombinant BCATs. One unit of BCAT activity was defined as the amount of enzyme required to produce 1 μmol of KG per min (for forward reaction) or the amount of enzyme required to produce 1 μmol of Glu per min (for reverse reaction). Kinetic parameters of each enzyme were calculated with GraphPad Prism version 9 (GraphPad Software) using nonlinear regression analysis.

### Data availability.

The data underlying this article are available in the article.

## References

[1] Atsumi S, Hanai T, Liao JC. 2008. Non-fermentative pathways for synthesis of branched-chain higher alcohols as biofuels. Nature 451:86–89. 10.1038/nature06450. 18172501

[2] Mainguet SE, Liao JC. 2010. Bioengineering of microorganisms for C3 to C5 alcohols production. Biotechnol J 5:1297–1308. 10.1002/biot.201000276. 21154669

[3] Cheng J, Jiang C. 2007. Analysis on process technology and market situation of isobutyl alcohol worldwide. Chem Ind 25:28–31.

[4] Amerine MA. 1980. The technology of wine making, 4th ed. AVI Technical Books Inc., Westport, CT, USA.

[5] Pires EJ, Teixeira JA, Brányik T, Vicente AA. 2014. Yeast: the soul of beer’s aroma. A review of flavour-active esters and higher alcohols produced by the brewing yeast. Appl Microbiol Biotechnol 98:1937–1949. 10.1007/s00253-013-5470-0. 24384752

[6] Wang Y-P, Wei X-Q, Guo X-W, Xiao D-G. 2020. Effect of the deletion of genes related to amino acid metabolism on the production of higher alcohols by *Saccharomyces cerevisiae*. Biomed Res Int 2020:6802512. 10.1155/2020/6802512. 33204707 PMC7665916

[7] Dan T, Ren W, Liu Y, Tian J, Chen H, Li T, Liu W. 2019. Volatile flavor compounds profile and fermentation characteristics of milk fermented by *Lactobacillus delbrueckii* subsp. bulgaricus. Front Microbiol 10:2183. 10.3389/fmicb.2019.02183. 31620117 PMC6759748

[8] Zhao G, Liu C, Hadiatullah H, Yao Y, Lu F. 2021. Effect of *Hericium erinaceus* on bacterial diversity and volatile flavor changes of soy sauce. LWT 139:110543. 10.1016/j.lwt.2020.110543.

[9] Watanabe M, Fukuda K, Asano K, Ohta S. 1990. Mutants of bakers’ yeasts producing a large amount of isobutyl alcohol or isoamyl alcohol, flavour components of bread. Appl Microbiol Biotechnol 34:154–159. 10.1007/BF00166772.

[10] Wang B-W, Shi A-Q, Tu R, Zhang X-L, Wang Q-H, Bai F-W. 2012. Branched-chain higher alcohols. Biotechnol China III Biofuels Bioenergy 128:101–118. 10.1007/10_2011_121. 22109725

[11] Olson ES, Sharma RK, Aulich TR. 2004. Higher-alcohols biorefinery, p 913–932. *In* Finkelstein M, McMillan JD, Davison BH, Evans B (ed), Proceedings of the Twenty-Fifth Symposium on Biotechnology for Fuels and Chemicals. Humana Press, Totowa, NJ.

[12] Hazelwood LA, Daran JM, Van Maris AJ, Pronk JT, Dickinson JR. 2008. The Ehrlich pathway for fusel alcohol production: a century of research on *Saccharomyces cerevisiae* metabolism. Appl Environ Microbiol 74:2259–2266. 10.1128/AEM.02625-07. 18281432 PMC2293160

[13] Hohmann S. 2002. Osmotic adaptation in yeast-control of the yeast osmolyte system. Int Rev Cytol 215:149–187. 10.1016/s0074-7696(02)15008-x. 11952227

[14] Porro D, Gasser B, Fossati T, Maurer M, Branduardi P, Sauer M, Mattanovich D. 2011. Production of recombinant proteins and metabolites in yeasts. Appl Microbiol Biotechnol 89:939–948. 10.1007/s00253-010-3019-z. 21125266

[15] Ryan D, Kohlhaw B. 1974. Subcellular localization of isoleucine-valine biosynthetic enzymes in yeast. J Bacteriol 120:631–637. 10.1128/jb.120.2.631-637.1974. 4616942 PMC245821

[16] Baichwal VR, Cunningham TS, Gatzek PR, Kohlhaw GB. 1983. Leucine biosynthesis in yeast. Curr Genet 7:369–377. 10.1007/BF00445877. 24173418

[17] Kispal G, Steiner H, Court DA, Rolinski B, Lill R. 1996. Mitochondrial and cytosolic branched-chain amino acid transaminases from yeast, homologs of the myc oncogene-regulated Eca39 protein. J Biol Chem 271:24458–24464. 10.1074/jbc.271.40.24458. 8798704

[18] Lilly M, Bauer FF, Styger G, Lambrechts MG, Pretorius IS. 2006. The effect of increased branched-chain amino acid transaminase activity in yeast on the production of higher alcohols and on the flavour profiles of wine and distillates. FEMS Yeast Res 6:726–743. 10.1111/j.1567-1364.2006.00057.x. 16879424

[19] Styger G, Jacobson D, Prior BA, Bauer FF. 2013. Genetic analysis of the metabolic pathways responsible for aroma metabolite production by *Saccharomyces cerevisiae*. Appl Microbiol Biotechnol 97:4429–4442. 10.1007/s00253-012-4522-1. 23111598

[20] Okada K, Hirotsu K, Sato M, Hayashi H, Kagamiyama H. 1997. Three-dimensional structure of *Escherichia coli* branched-chain amino acid aminotransferase at 2.5 Å resolution. J Biochem 121:637–641. 10.1093/oxfordjournals.jbchem.a021633. 9163511

[21] Takpho N, Watanabe D, Takagi H. 2018. Valine biosynthesis in *Saccharomyces cerevisiae* is regulated by the mitochondrial branched-chain amino acid aminotransferase Bat1. Microb Cell 5:293–299. 10.15698/mic2018.06.637. 29850466 PMC5972033

[22] Eden A, Simchen G, Benvenisty N. 1996. Two yeast homologs of ECA39, a target for c-Myc regulation, code for cytosolic and mitochondrial branched-chain amino acid aminotransferases. J Biol Chem 271:20242–20245. 10.1074/jbc.271.34.20242. 8702755

[23] Chen X, Nielsen KF, Borodina I, Kielland-Brandt MC, Karhumaa K. 2011. Increased isobutanol production in *Saccharomyces cerevisiae* by overexpression of genes in valine metabolism. Biotechnol Biofuels 4:21. 10.1186/1754-6834-4-21. 21798060 PMC3162486

[24] Eden A, Van Nedervelde L, Drukker M, Benvenisty N, Debourg A. 2001. Involvement of branched-chain amino acid aminotransferases in the production of fusel alcohols during fermentation in yeast. Appl Microbiol Biotechnol 55:296–300. 10.1007/s002530000506. 11341309

[25] Hammer SK, Avalos JL. 2017. Uncovering the role of branched-chain amino acid transaminases in *Saccharomyces cerevisiae* isobutanol biosynthesis. Metab Eng 44:302–312. 10.1016/j.ymben.2017.10.001. 29037781

[26] Koonthongkaew J, Toyokawa Y, Ohashi M, Large CRL, Dunham MJ, Takagi H. 2020. Effect of the Ala234Asp replacement in mitochondrial branched-chain amino acid aminotransferase on the production of BCAAs and fusel alcohols in yeast. Appl Microbiol Biotechnol 104:7915–7925. 10.1007/s00253-020-10800-y. 32776205

[27] Zhang C-Y, Qi Y-N, Ma H-X, Li W, Dai L-H, Xiao D-G. 2015. Decreased production of higher alcohols by *Saccharomyces cerevisiae* for Chinese rice wine fermentation by deletion of Bat aminotransferases. J Ind Microbiol Biotechnol 42:617–625. 10.1007/s10295-015-1583-z. 25616436

[28] Zhang A, Li Y, Gao Y, Jin H. 2016. Increasing isobutanol yield by double-gene deletion of PDC6 and LPD1 in *Saccharomyces cerevisiae*. Chinese J Chem Eng 24:1074–1079. 10.1016/j.cjche.2016.04.004.

[29] Knerr I, Colombo R, Urquhart J, Morais A, Merinero B, Oyarzabal A, Pérez B, Jones SA, Perveen R, Preece MA, Rogers Y, Treacy EP, Mayne P, Zampino G, MacKinnon S, Wassmer E, Yue WW, Robinson I, Rodríguez-Pombo P, Olpin SE, Banka S. 2019. Expanding the genetic and phenotypic spectrum of branched-chain amino acid transferase 2 deficiency. J Inherit Metab Dis 42:809–817. 10.1002/jimd.12135. 31177572

[30] Lei MZ, Li XX, Zhang Y, Li JT, Zhang F, Wang YP, Yin M, Qu J, Lei QY. 2020. Acetylation promotes BCAT2 degradation to suppress BCAA catabolism and pancreatic cancer growth. Signal Transduct Target Ther 5:70. 10.1038/s41392-020-0168-0. 32467562 PMC7256045

[31] Li JT, Yin M, Wang D, Wang J, Lei MZ, Zhang Y, Liu Y, Zhang L, Zou SW, Hu LP, Zhang ZG, Wang YP, Wen WY, Lu HJ, Chen ZJ, Su D, Lei QY. 2020. BCAT2-mediated BCAA catabolism is critical for development of pancreatic ductal adenocarcinoma. Nat Cell Biol 22:167–174. 10.1038/s41556-019-0455-6. 32029896

[32] Wang XL, Li CJ, Xing Y, Yang YH, Jia JP. 2015. Hypervalinemia and hyperleucine-isoleucinemia caused by mutations in the branched-chain-amino-acid aminotransferase gene. J Inherit Metab Dis 38:855–861. 10.1007/s10545-015-9814-z. 25653144

[33] Wu JY, Kao HJ, Li SC, Stevens R, Hillman S, Millington D, Chen YT. 2004. ENU mutagenesis identifies mice with mithochondrial branched-chain aminotransferase deficiency resembling human maple syrup urine disease. J Clin Invest 113:434–440. 10.1172/JCI19574. 14755340 PMC324540

[34] Espinosa-Cantú A, Ascencio D, Herrera-Basurto S, Xu J, Roguev A, Krogan NJ, DeLuna A. 2018. Protein moonlighting revealed by noncatalytic phenotypes of yeast enzymes. Genetics 208:419–431. 10.1534/genetics.117.300377. 29127264 PMC5753873

[35] Kingsbury JM, Sen ND, Cardenas ME. 2015. Branched-chain aminotransferases control TORC1 signaling in *Saccharomyces cerevisiae*. PLoS Genet 11:e1005714. 10.1371/journal.pgen.1005714. 26659116 PMC4684349

[36] Reetz MT, Carballeira JD, Vogel A. 2006. Iterative saturation mutagenesis on the basis of B factors as a strategy for increasing protein thermostability. Angew Chem 118:7909–7915. 10.1002/ange.200602795. 17075931

[37] Bloom JD, Labthavikul ST, Otey CR, Arnold FH. 2006. Protein stability promotes evolvability. Proc Natl Acad Sci USA 103:5869–5874. 10.1073/pnas.0510098103. 16581913 PMC1458665

[38] Korendovych IV. 2018. Rational and semirational protein design. Methods Mol Biol 1685:15–23. 10.1007/978-1-4939-7366-8_2. 29086301 PMC5912912

[39] Schoondermark-Stolk SA, Tabernero M, Chapman J, Ter Schure EG, Verrips CT, Verkleij AJ, Boonstra J. 2005. Bat2p is essential in *Saccharomyces cerevisiae* for fusel alcohol production on the non-fermentable carbon source ethanol. FEMS Yeast Res 5:757–766. 10.1016/j.femsyr.2005.02.005. 15851104

[40] Davoodi J, Drown PM, Bledsoe RK, Wallin R, Reinhart GD, Hutson SM. 1998. Overexpression and characterization of the human mitochondrial and cytosolic branched-chain aminotransferases. J Biol Chem 273:4982–4989. 10.1074/jbc.273.9.4982. 9478945

[41] Prohl C, Kispal G, Lill R. 2000. Branched-chain-amino-acid transaminases of yeast *Saccharomyces cerevisiae*. Methods Enzymol 324:365–375. 10.1016/s0076-6879(00)24246-8. 10989445

[42] Goto M, Miyahara I, Hayashi H, Kagamiyama H, Hirotsu K. 2003. Crystal structures of branched-chain amino acid aminotransferase complexed with glutamate and glutarate: true reaction intermediate and double substrate recognition of the enzyme. Biochemistry 42:3725–3733. 10.1021/bi026722f. 12667063

[43] Hu L-Y, Boxer PA, Kesten SR, Lei HJ, Wustrow DJ, Moreland DW, Zhang L, Ahn K, Ryder TR, Liu X, Rubin JR, Fahnoe K, Carroll RT, Dutta S, Fahnoe DC, Probert AW, Roof RL, Rafferty MF, Kostlan CR, Scholten JD, Hood M, Ren X-D, Schielke GP, Su T-Z, Taylor CP, Mistry A, McConnell P, Hasemann C, Ohren J. 2006. The design and synthesis of human branched-chain amino acid aminotransferase inhibitors for treatment of neurodegenerative diseases. Bioorg Med Chem Lett 16:2337–2340. 10.1016/j.bmcl.2005.07.058. 16143519

[44] Sperringer JE, Addington A, Hutson SM. 2017. Branched-chain amino acids and brain metabolism. Neurochem Res 42:1697–1709. 10.1007/s11064-017-2261-5. 28417264

[45] Eliot AC, Kirsch JF. 2004. Pyridoxal phosphate enzymes: mechanistic, structural, and evolutionary considerations. Annu Rev Biochem 73:383–415. 10.1146/annurev.biochem.73.011303.074021. 15189147

[46] Dawson RMC, Elliott DC, Elliott WH, Jones KM. 2002. Data for biochemical research, vol 3. Clarendon Press, London, United Kingdom.

[47] Colón M, Hernández F, López K, Quezada H, González J, López G, Aranda C, González A. 2011. *Saccharomyces cerevisiae* Bat1 and Bat2 aminotransferases have functionally diverged from the ancestral-like *Kluyveromyces lactis* orthologous enzyme. PLoS One 6:e16099. 10.1371/journal.pone.0016099. 21267457 PMC3022659

[48] González J, López G, Argueta S, Escalera-Fanjul X, El Hafidi M, Campero-Basaldua C, Strauss J, Riego-Ruiz L, González A. 2017. Diversification of transcriptional regulation determines subfunctionalization of paralogous branched chain aminotransferases in the yeast *Saccharomyces cerevisiae*. Genetics 207:975–991. 10.1534/genetics.117.300290. 28912343 PMC5676234

[49] Park S-H, Kim S, Hahn J-S. 2014. Metabolic engineering of *Saccharomyces cerevisiae* for the production of isobutanol and 3-methyl-1-butanol. Appl Microbiol Biotechnol 98:9139–9147. 10.1007/s00253-014-6081-0. 25280745

[50] Marques SM, Bednar D, Damborsky J. 2018. Computational study of protein-ligand unbinding for enzyme engineering. Front Chem 6:650. 10.3389/fchem.2018.00650. 30671430 PMC6331733

[51] Agarwal PK. 2005. Role of protein dynamics in reaction rate enhancement by enzymes. J Am Chem Soc 127:15248–15256. 10.1021/ja055251s. 16248667

[52] Agarwal PK. 2006. Enzymes: an integrated view of structure, dynamics and function. Microb Cell Fact 5:2–12. 10.1186/1475-2859-5-2. 16409630 PMC1379655

[53] Eisenmesser EZ, Bosco DA, Akke M, Kern D. 2002. Enzyme dynamics during catalysis. Science 295:1520–1523. 10.1126/science.1066176. 11859194

[54] Fernandes HS, Ramos MJ, Cerqueira N. 2017. The catalytic mechanism of the pyridoxal-5′-phosphate-dependent enzyme, histidine decarboxylase: a computational study. Chemistry 23:9162–9173. 10.1002/chem.201701375. 28613002

[55] Reuveni S, Urbakh M, Klafter J. 2014. Role of substrate unbinding in Michaelis–Menten enzymatic reactions. Proc Natl Acad Sci USA 111:4391–4396. 10.1073/pnas.1318122111. 24616494 PMC3970482

[56] Avalos JL, Fink GR, Stephanopoulos G. 2013. Compartmentalization of metabolic pathways in yeast mitochondria improves the production of branched-chain alcohols. Nat Biotechnol 31:335–341. 10.1038/nbt.2509. 23417095 PMC3659820

[57] Hammer SK, Zhang Y, Avalos JL. 2020. Mitochondrial compartmentalization confers specificity to the 2-ketoacid recursive pathway: increasing isopentanol production in *Saccharomyces cerevisiae*. ACS Synth Biol 9:546–555. 10.1021/acssynbio.9b00420. 32049515

[58] Schwede T, Kopp J, Guex N, Peitsch MC. 2003. SWISS-MODEL: an automated protein homology-modeling server. Nucleic Acids Res 31:3381–3385. 10.1093/nar/gkg520. 12824332 PMC168927

[59] Laskowski RA, MacArthur MW, Moss DS, Thornton JM. 1993. PROCHECK: a program to check the stereochemical quality of protein structures. J Appl Crystallogr 26:283–291. 10.1107/S0021889892009944.

[60] Laskowski RA, Jabłońska J, Pravda L, Vařeková RS, Thornton JM. 2018. PDBsum: structural summaries of PDB entries. Protein Sci 27:129–134. 10.1002/pro.3289. 28875543 PMC5734310

[61] Pettersen EF, Goddard TD, Huang CC, Couch GS, Greenblatt DM, Meng EC, Ferrin TE. 2004. UCSF Chimera: a visualization system for exploratory research and analysis. J Comput Chem 25:1605–1612. 10.1002/jcc.20084. 15264254

[62] Shapovalov MV, Dunbrack RL. 2011. A smoothed backbone-dependent rotamer library for proteins derived from adaptive kernel density estimates and regressions. Structure 19:844–858. 10.1016/j.str.2011.03.019. 21645855 PMC3118414

[63] Wang J, Wang W, Kollman PA, Case DA. 2006. Automatic atom type and bond type perception in molecular mechanical calculations. J Mol Graph Model 25:247–260. 10.1016/j.jmgm.2005.12.005. 16458552

[64] Jubb HC, Higueruelo AP, Ochoa-Montaño B, Pitt WR, Ascher DB, Blundell TL. 2017. Arpeggio: a web server for calculating and visualising interatomic interactions in protein structures. J Mol Biol 429:365–371. 10.1016/j.jmb.2016.12.004. 27964945 PMC5282402

[65] Frisch MJ, Trucks GW, Schlegel HB, Scuseria GE, Robb MA, Cheeseman JR, Scalmani G, Barone V, Mennucci B, Petersson GA. 2009. Gaussian 09. Gaussian, Inc., Wallingford, CT, USA.

[66] Becke AD. 1992. Density‐functional thermochemistry. I. The effect of the exchange‐only gradient correction. J Chem Phys 96:2155–2160. 10.1063/1.462066.

[67] Hehre WJ, Ditchfield R, Pople JA. 1972. Self-consistent molecular orbital methods. XII. Further extensions of Gaussian-type basis sets for use in molecular orbital studies of organic molecules. J Chem Phys 56:2257–2261. 10.1063/1.1677527.

[68] Lee C, Yang W, Parr RG. 1988. Development of the Colle-Salvetti correlation-energy formula into a functional of the electron density. Phys Rev B Condens Matter 37:785–789. 10.1103/physrevb.37.785. 9944570

[69] Trott O, Olson AJ. 2010. Software news and update. AutoDock Vina: improving the speed and accuracy of docking with a new scoring function, efficient optimization, and multithreading. J Comput Chem 31:455–461. 19499576 10.1002/jcc.21334 PMC3041641

[70] Sanner MF. 1999. Python: a programming language for software integration and development. J Mol Graph Model 17:57–61. 10660911

[71] Laskowski RA, Swindells MB. 2011. LigPlot+: multiple ligand-protein interaction diagrams for drug discovery. J Chem Inf Model 51:2778–2786. 10.1021/ci200227u. 21919503

[72] Parthiban V, Gromiha MM, Schomburg D. 2006. CUPSAT: prediction of protein stability upon point mutations. Nucleic Acids Res 34:W239–W242. 10.1093/nar/gkl190. 16845001 PMC1538884

[73] Pires DEV, Blundell TL, Ascher DB. 2016. mCSM-lig: quantifying the effects of mutations on protein-small molecule affinity in genetic disease and emergence of drug resistance. Sci Rep 6:29575–29578. 10.1038/srep29575. 27384129 PMC4935856

[74] Inoue H, Nojima H, Okayama H. 1990. High efficiency transformation of Escherichia coli with plasmids. Gene 96:23–28. 10.1016/0378-1119(90)90336-p. 2265755

[75] Burke D, Dawson D, Stearns T. 2000. Methods in yeast genetics: a Cold Spring Harbor Laboratory course manual. Cold Spring Harbor Laboratory Press, Plainview, NY, USA.

[76] St-Jacques AD, Eyahpaise MÈC, Chica RA. 2019. Computational design of multisubstrate enzyme specificity. ACS Catal 9:5480–5485. 10.1021/acscatal.9b01464.

