# Supplementary material for: Improvement of Fusel Alcohol Production by Engineering of the Yeast Branched-Chain Amino Acid Aminotransaminase
Source: Appl Environ Microbiol. 2022 Jun 14;88(13):e00557-22. doi: 10.1128/aem.00557-22 (PMC9275217; doi:10.1128/aem.00557-22)
Supplement: Supplemental file 1 — Tables S1 to S4 and Fig. S1 to S6. Download aem.00557-22-s0001.pdf, PDF file, 1.5 MB [file aem.00557-22-s0001.pdf]

Supplementary Material

Improvement of Fusel Alcohol Production by Engineering of the Yeast Branched-chain Amino Acid Aminotransaminase

Jirasin Koonthongkaew,[a] Nontawat Ploysongsri,[b] Yoichi Toyokawa,[a] Vithaya Ruangpornvisuti,[b] Hiroshi Takagi[a#]

[a]Division of Biological Science, Graduate School of Science and Technology, Nara Institute of Science and Technology, 8916-5 Takayama, Ikoma, Nara, Japan

[b]Department of Chemistry, Faculty of Science, Chulalongkorn University, 254 Phyathai Road, Patumwan, Bangkok 10330, Thailand

Running title: Fusel Alcohol Production by Engineering the Yeast BCAT

#Address correspondence to Hiroshi Takagi, hiro@bs.naist.jp

This file includes TABLE S1-S4, FIG S1-S6.

TABLE S1 Structural validation of Bat2 homology structure that was modeled from SWISS-MODEL

Protein	Quality check parameters											
	SWISS-MODEL parameters					Ramachandran Plot statistics				G-Factors		
	Template	Sequence identity (%)	QSQE	GMQE	QMEAN (Z-score)	Most favoured regions [A, B, L] (%)	Additional allowed regions [a, b, l, p] (%)	Genero -usly allowed regions [~a, ~b, ~l, ~p]	Dis-allowed region	Dihedral angles	Main-chain covalent forces	Overall average
Bat2	2abj.1.A	47.77	0.92	0.76	-0.96	90.5	8.9	0.2	0.5	-0.32	-0.01	-0.18

TABLE S2 Interactive amino acid residues for substrate-binding on Bat2

BCKAs	Bat2 active site residues
KIV	Phe30, Tyr71, Phe76, **Tyr142, Arg144,** Val156, **Lys202,** Thr240, Ala318
KIC	Phe30, Tyr71, Phe76, **Tyr142**, **Arg144**, Val156, Lys202, Tyr207, Thr240, Ala318
KMV	Phe76, **Tyr142**, **Arg144**, Tyr174, Lys202, Thr240, Ala318
**BCAAs**	
Val	Phe30, Tyr71, Phe76, Gly78, **Tyr142**, Val156, **Lys202**, Ala318
Leu	Phe30, Tyr71, **Tyr142**, Leu154, Val156, Tyr174, Thr240, Gly316, **Ala318**
Ile	Phe30, **Tyr71**, Phe76, Tyr142, Val156, Tyr174, Lys202, Tyr207, Thr240, Ala318

Residues represented by bold indicate hydrogen formation residues to each substrate.

TABLE S3 Docking-configurations of the substrates-Bat2 interactions

Ligand	Interaction residues of Bat2	Ligand and enzyme atom involved in H-bonding
**BCKAs**		
KIV	A: Tyr71, Val156<br>B: Phe30, Phe76, Tyr142, Arg144, Lys202, Thr240, Ala318, (PLP)	O1; Tyr142:OH (2.74 Å)<br>O1; Arg144:NH1 (2.80 Å)<br>O2; Lys202:NZ (3.25 Å)
KIC	A: Tyr71, Val156<br>B: Phe30, Phe76, Tyr142, Arg144, Lys202, Tyr207, Thr240, Ala318, (PLP)	O1; Tyr142:OH (3.16 Å)<br>O1; Arg144:NH1 (2.86 Å)<br>O2; PLP:O3 (3.09 Å)
KMV	A: -<br>B: Phe76, Tyr142, Arg144, Tyr174, Lys202, Thr240, Ala318, (PLP)	O1; Tyr142:OH (3.15 Å)<br>O1; Arg144:NH1 (2.87 Å)
**BCAAs**		
Val	A: Tyr71, Val156<br>B: Phe30, Phe76, Gly78, Tyr142, Lys202, Ala318, (PLP)	O1; Lys202:NZ (3.27 Å)<br>N; Tyr142:OH (2.99 Å)
Leu	A: Tyr71, Leu154, Val156<br>B: Phe30, Tyr142, Tyr174, Thr240, Gly316, Ala318, (PLP)	O1; Ala318:N (3.05 Å)<br>N; Tyr142:OH (3.06 Å)
Ile	A: Tyr71, Val156<br>B: Phe30, Phe76, Tyr142, Tyr174, Lys202, Tyr207, Thr240, Ala318, (PLP)	O2; PLP:O3 (3.11 Å)<br>N; Tyr71:OH (3.02 Å)

TABLE S4 Predicted effect of amino acid substitutions on protein stability

Original amino acid	Mutated amino acid	Overall Stability	Torsion	Predicted ΔΔG (kcal/mol)
Phe30	Cys	Stabilizing	Unfavorable	0.06
Phe76	Cys	Stabilizing	Favorable	2.89
Glu77	Thr	Stabilizing	Favorable	2.43
	Gln	Stabilizing	Unfavorable	1.66
	Lys	Stabilizing	Unfavorable	4.85
	Asp	Stabilizing	Unfavorable	1.73
	His	Stabilizing	Favorable	3.93
Gly78	Val	Stabilizing	Unfavorable	0.53
	Leu	Stabilizing	Unfavorable	4.17
	Ile	Stabilizing	Unfavorable	1.31
	Met	Stabilizing	Unfavorable	1.95
	Thr	Stabilizing	Unfavorable	1.72
	Phe	Stabilizing	Favorable	1.33
	Lys	Stabilizing	Unfavorable	1.58
	Asn	Stabilizing	Favorable	1.65
	Glu	Stabilizing	Unfavorable	5.54
	Arg	Stabilizing	Unfavorable	3.06
	His	Stabilizing	Favorable	4.66
Leu154	Gly	Stabilizing	Unfavorable	0.76
	Met	Stabilizing	Unfavorable	1.97
	Thr	Stabilizing	Unfavorable	0.84
	Gln	Stabilizing	Favorable	0.94
	Lys	Stabilizing	Unfavorable	5.79
	Glu	Stabilizing	Unfavorable	2.39
	Arg	Stabilizing	Unfavorable	4.92
	His	Stabilizing	Favorable	3.21
Gly155	Ala	Stabilizing	Favorable	0.08
	Pro	Stabilizing	Favorable	1.21
	Thr	Stabilizing	Unfavorable	0.59
	Phe	Stabilizing	Unfavorable	0.79
	Gln	Stabilizing	Unfavorable	0.74
	Cys	Stabilizing	Unfavorable	0.62
Tyr174	Gly	Stabilizing	Unfavorable	1.14
	Ala	Stabilizing	Unfavorable	1.21
	Val	Stabilizing	Unfavorable	0.69

	Leu	Stabilizing	Favorable	0.74
	Ile	Stabilizing	Unfavorable	2.84
	Met	Stabilizing	Favorable	2.72
	Pro	Stabilizing	Unfavorable	0.01
	Ser	Stabilizing	Favorable	1.23
	Thr	Stabilizing	Favorable	1.37
	Gln	Stabilizing	Favorable	0.98
	Lys	Stabilizing	Favorable	1.67
	Asn	Stabilizing	Favorable	1.44
	Cys	Stabilizing	Unfavorable	3.13
	Glu	Stabilizing	Unfavorable	2.25
	Asp	Stabilizing	Favorable	2.0
	Arg	Stabilizing	Unfavorable	0.05
	His	Stabilizing	Favorable	0.53
Thr240	Pro	Stabilizing	Unfavorable	0.74
	Trp	Stabilizing	Unfavorable	0.4
	Ser	Stabilizing	Unfavorable	0.62
	Phe	Stabilizing	Favorable	0.1
	Gln	Stabilizing	Unfavorable	0.06
	Cys	Stabilizing	No change	1.34
Gly316	Leu	Stabilizing	Unfavorable	0.04
	Ile	Stabilizing	Unfavorable	0.87
	Trp	Stabilizing	Unfavorable	3.44
	Ser	Stabilizing	Favorable	0.44
	Thr	Stabilizing	Unfavorable	1.4
	Gln	Stabilizing	Unfavorable	3.03
	Lys	Stabilizing	Unfavorable	2.35
	Tyr	Stabilizing	Favorable	1.02
	Asn	Stabilizing	Unfavorable	2.31
	Glu	Stabilizing	Unfavorable	2.49
	Asp	Stabilizing	Unfavorable	4.0
	Arg	Stabilizing	Unfavorable	3.09
	His	Stabilizing	Favorable	1.17
Ala318	Val	Stabilizing	Favorable	0.95
	Leu	Stabilizing	Favorable	0.09
	Ile	Stabilizing	Favorable	0.75
	Pro	Stabilizing	Unfavorable	1.37
	Trp	Stabilizing	Favorable	1.63
	Gln	Stabilizing	Favorable	0.15
	Tyr	Stabilizing	Favorable	0.02
	Cys	Stabilizing	Unfavorable	0.38

	His	Stabilizing	Unfavorable	0.17
Ala319	Val	Stabilizing	Unfavorable	0.1
	Pro	Stabilizing	Unfavorable	0.59
	Trp	Stabilizing	Favorable	0.48
	Thr	Stabilizing	Favorable	0.74
	Arg	Stabilizing	No change	0.06

ΔΔG was calculated from CUPSAT. The positive and negative in ΔΔG value indicate increased and decreased protein stability, respectively.

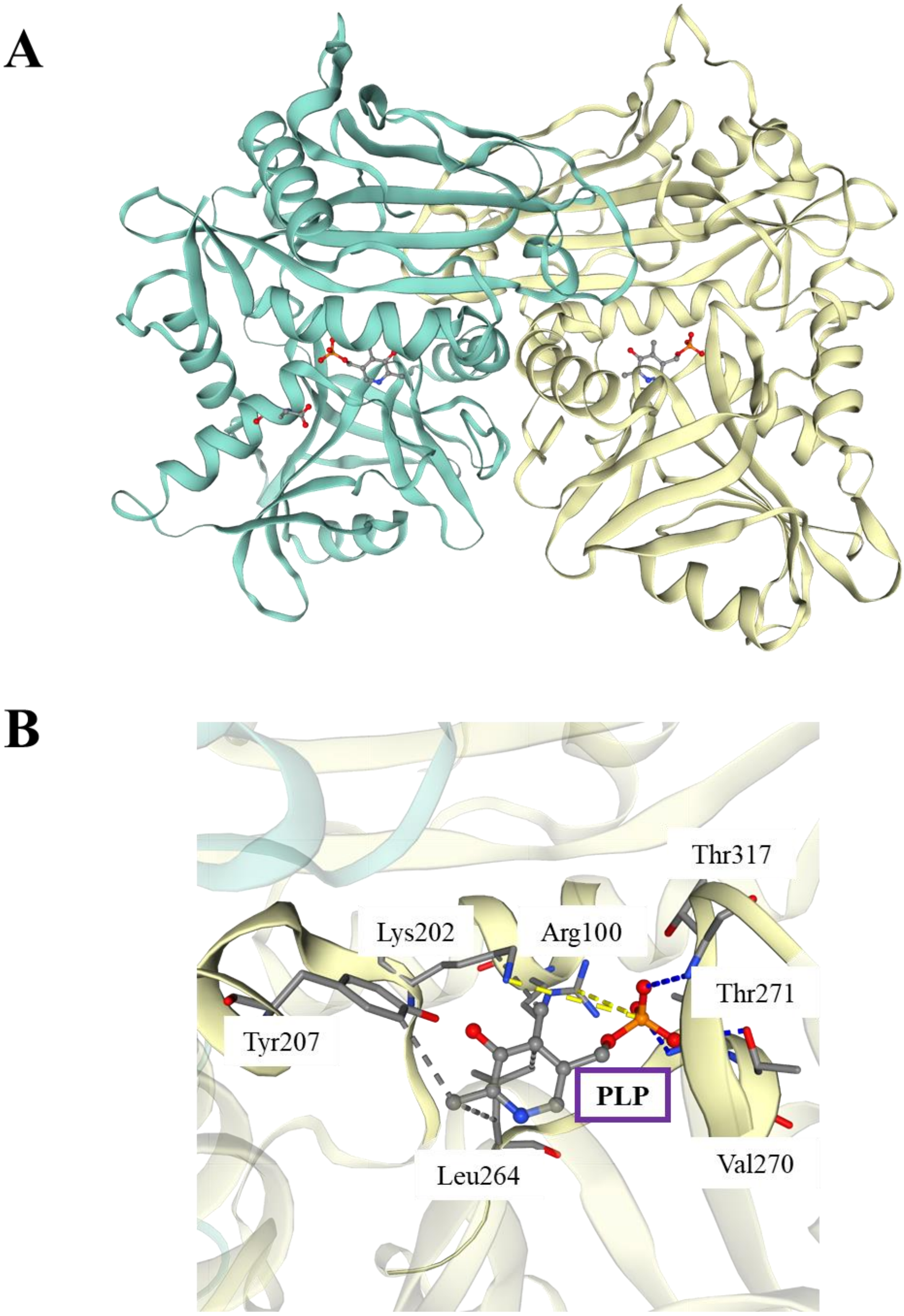

FIG S1 Modeled homology structure of Bat2 from SWISS-MODEL. Homo-dimer of Bat2 and the active site with PLP incorporated (A) and PLP-interactive residues of Bat2 that interact with PLP (B). A difference in ribbon color represents a different chain of Bat2, forming a homo-dimer structure (chain A, yellow and chain B, green-blue color, respectively).

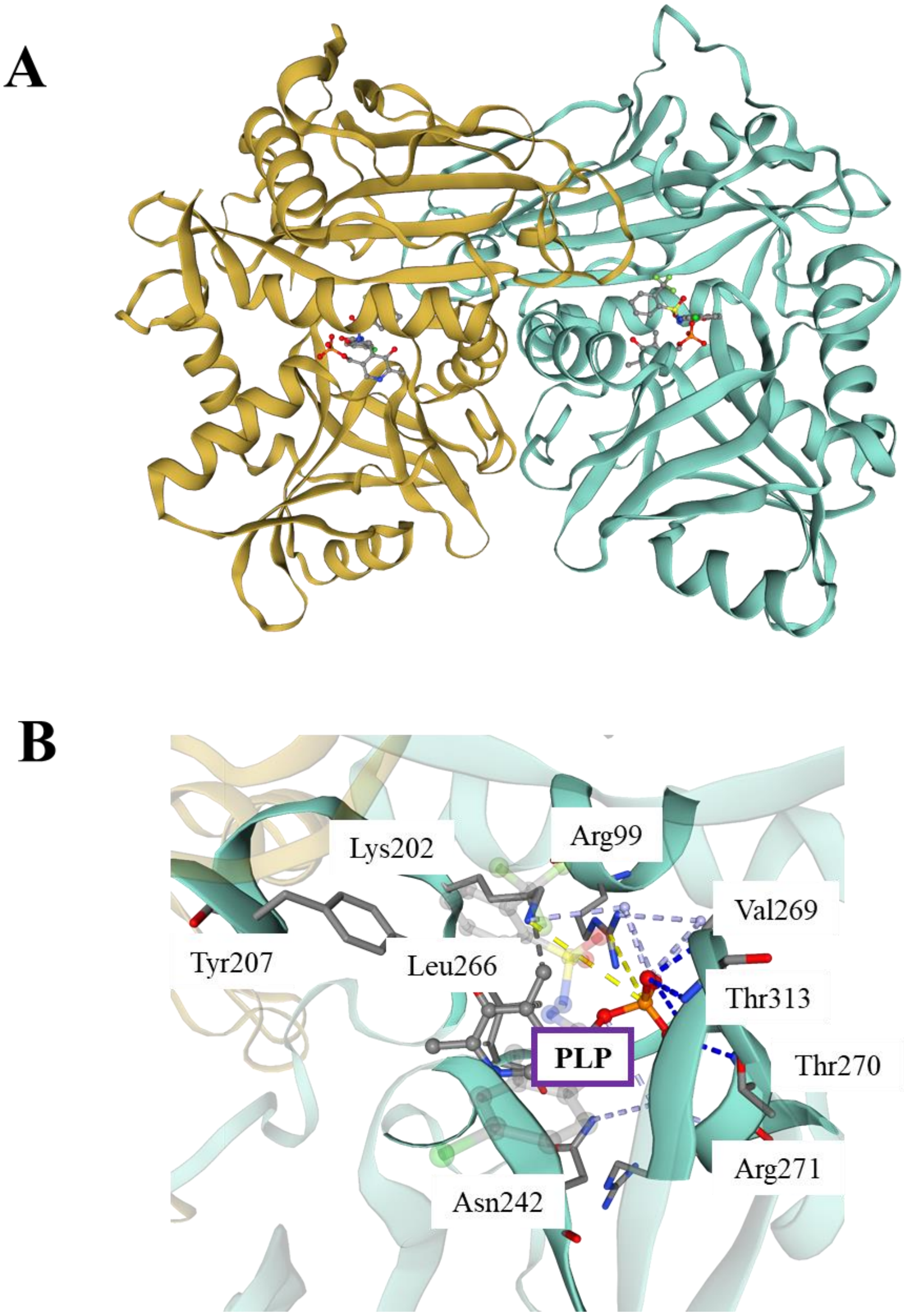

FIG S2 Crystal structure of human cytosolic branched-chain amino acid transaminase (hBCATc). The structure was obtained from SWISS-MODEL with SMTL ID: 2abj.1. (A) ribbon representative structure (chain A, yellow and chain B, blue-green) and (B) PLP-interactive residues.

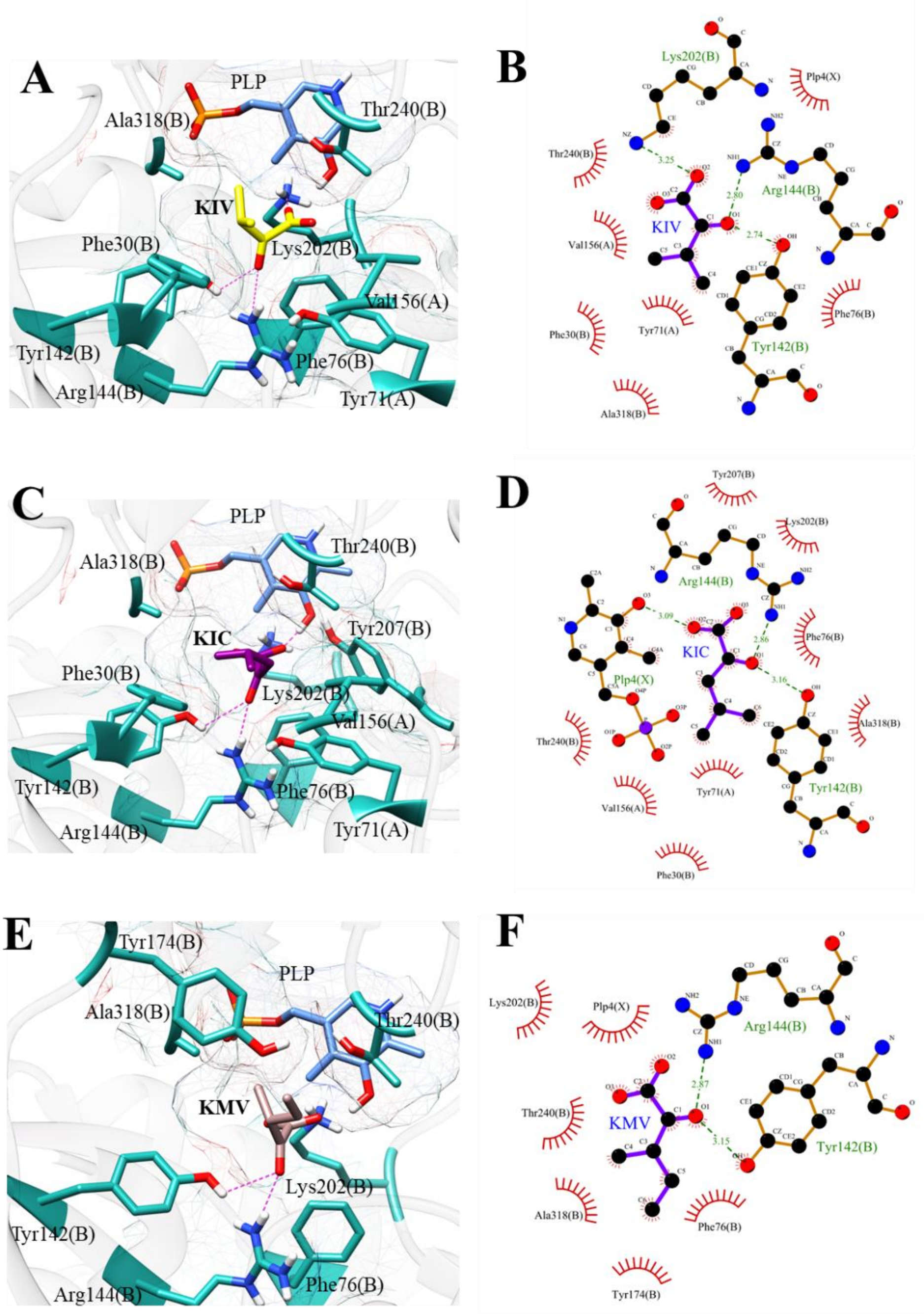

FIG S3 Binding of substrates [KIV, (A) and (B); KIC (C) and (D); KMV (E) and (F)] in the active site in Bat2. (A), (C) and (D) are 3D plots, and (B), (D), and (F) are 2D plots. Red and blue colors represent the surfaces of chains A and B, respectively. The sticks with different colors represent each substrate that binds to Bat2 (KIV, hot pink stick; KIC, green stick; KMV, gray stick).

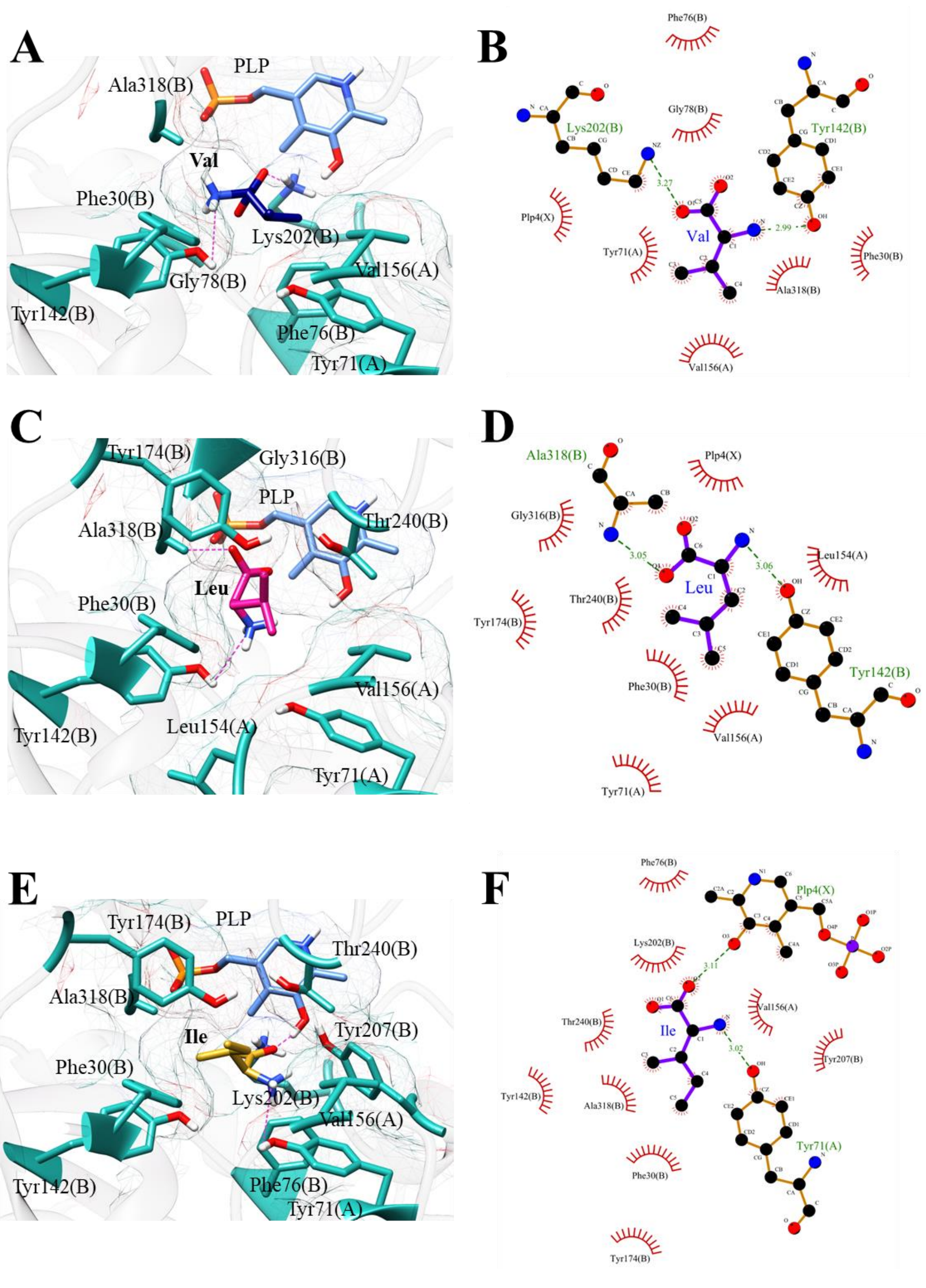

FIG S4 Binding of substrates [Val, (A) and (B); Leu (C) and (D); Ile (E) and (F)] in the active site in Bat2. (A), (C) and (D) are 3D plots, and (B), (D), and (F) are 2D plots. Red and blue colors represent the surfaces of chains A and B, respectively. The sticks with different colors represent each substrate that binds to Bat2 (Val, yellow stick; Leu, magenta stick; Ile, cyan stick; KG, purple stick; and Glu, gold stick).

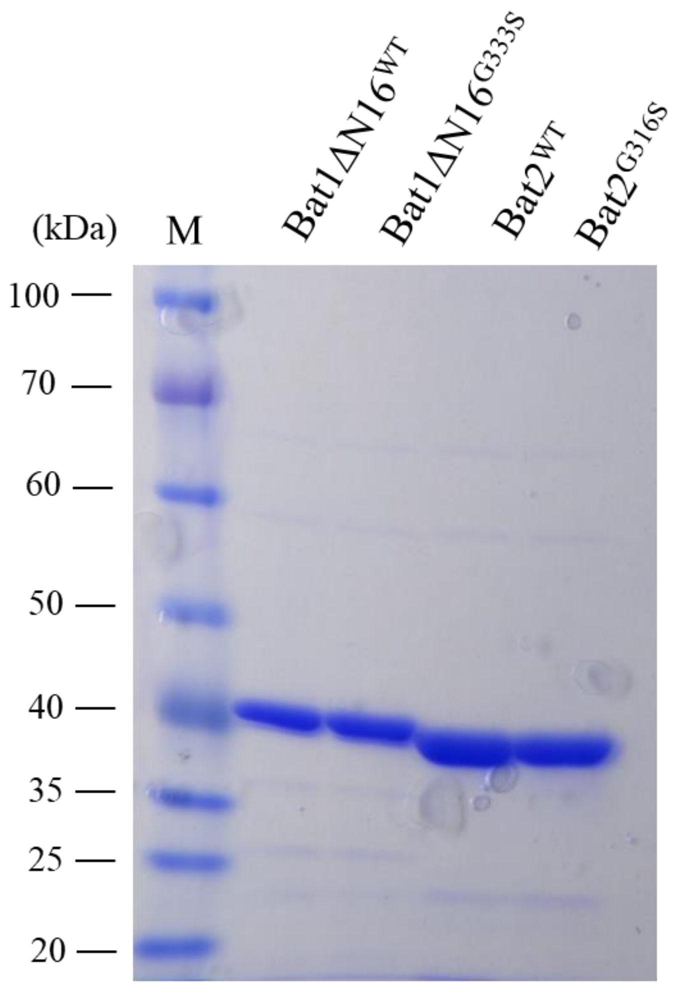

FIG S5 SDS-polyacrylamide gel electrophoresis of the purified recombinant BCATs. Lane M: Molecular mass standards, WT-Bat1ΔN16, Bat1ΔN16^{G333S}, WT-Bat2, and Bat2^{G316S}: recombinant Bat1 (without amino acid residues 1-16 at N-terminus), G333S variant of recombinant Bat1 (without amino acid residues 1-16 at N-terminus), recombinant Bat2, and G316S variant of the recombinant Bat2.

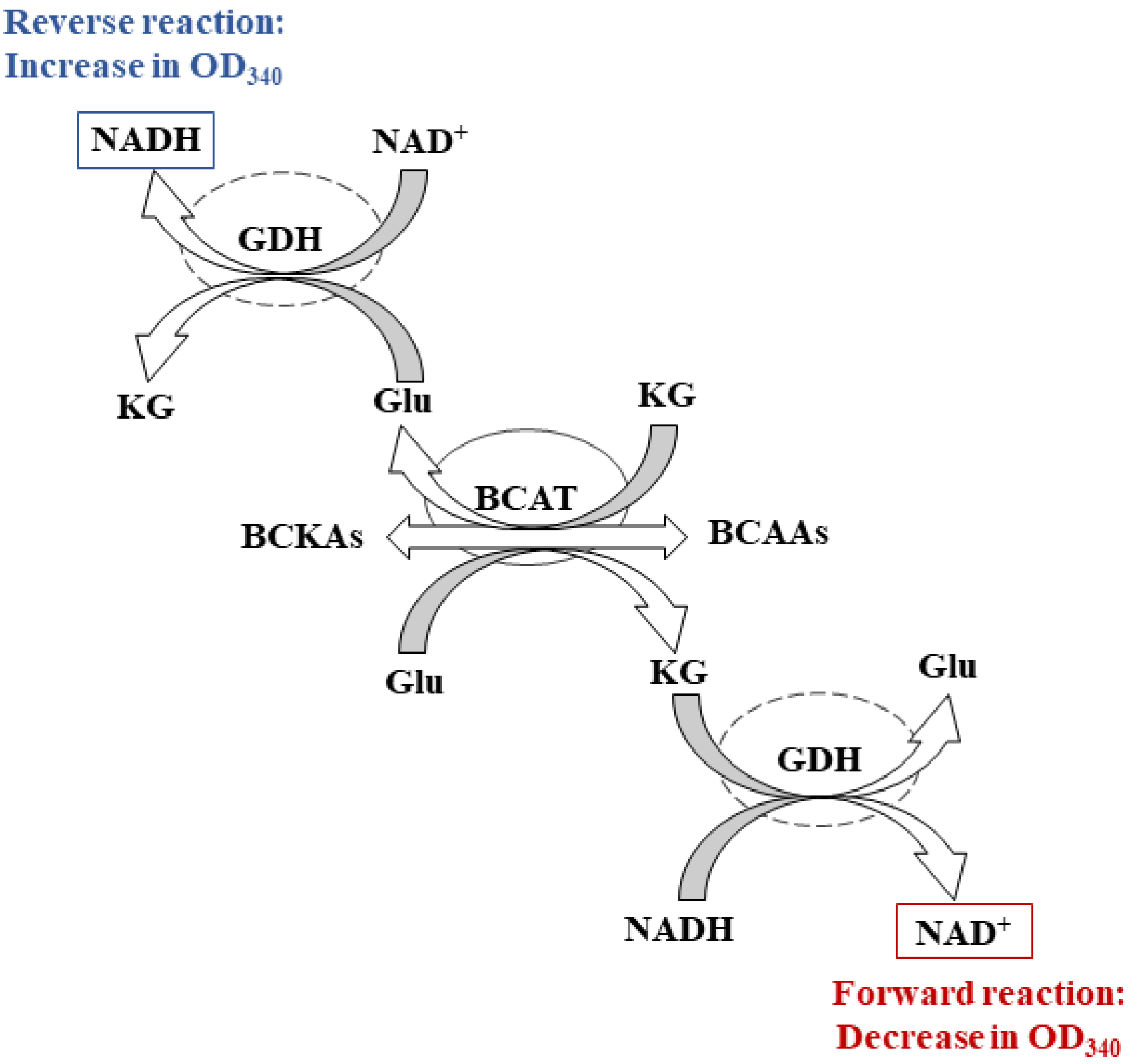

FIG S6 Schematic branched-chain amino acid transaminases (BCATs) assay. Glutamate dehydrogenase (GDH) couples the bi-directional transamination reaction of the yeast Bat1 and Bat2. GDH can utilize the product from forward-reaction (α-ketoglutarate, KG) and reverse-reaction (glutamate, Glu), which leads to degradation of NADH to NAD^+ (forward-reaction) or generation of NADH from NAD^+ (reverse-reaction). The generation or degradation of NADH level was measured the absorbance at 340 nm.
